# The study of atmospheric ice-nucleating particles via microfluidically generated droplets

**DOI:** 10.1007/s10404-018-2069-x

**Published:** 2018-04-24

**Authors:** Mark D. Tarn, Sebastien N. F. Sikora, Grace C. E. Porter, Daniel O’Sullivan, Mike Adams, Thomas F. Whale, Alexander D. Harrison, Jesús Vergara-Temprado, Theodore W. Wilson, Jung-uk Shim, Benjamin J. Murray

**Affiliations:** 1 School of Earth and Environment, University of Leeds, Leeds, LS2 9JT UK; 2 School of Physics and Astronomy, University of Leeds, Leeds, LS2 9JT UK; 3 Present Address: Institute for Atmospheric and Climate Science, ETH Zürich, Universitätstrasse 16, 8092 Zurich, Switzerland; 4 Present Address: Owlstone Medical Ltd., 127 Science Park, Cambridge, CB4 0GD UK

**Keywords:** Ice-nucleating particles (INPs), Atmospheric sampling, Droplets, Immersion mode freezing

## Abstract

Ice-nucleating particles (INPs) play a significant role in the climate and hydrological cycle by triggering ice formation in supercooled clouds, thereby causing precipitation and affecting cloud lifetimes and their radiative properties. However, despite their importance, INP often comprise only 1 in 10^3^–10^6^ ambient particles, making it difficult to ascertain and predict their type, source, and concentration. The typical techniques for quantifying INP concentrations tend to be highly labour-intensive, suffer from poor time resolution, or are limited in sensitivity to low concentrations. Here, we present the application of microfluidic devices to the study of atmospheric INPs via the simple and rapid production of monodisperse droplets and their subsequent freezing on a cold stage. This device offers the potential for the testing of INP concentrations in aqueous samples with high sensitivity and high counting statistics. Various INPs were tested for validation of the platform, including mineral dust and biological species, with results compared to literature values. We also describe a methodology for sampling atmospheric aerosol in a manner that minimises sampling biases and which is compatible with the microfluidic device. We present results for INP concentrations in air sampled during two field campaigns: (1) from a rural location in the UK and (2) during the UK’s annual Bonfire Night festival. These initial results will provide a route for deployment of the microfluidic platform for the study and quantification of INPs in upcoming field campaigns around the globe, while providing a benchmark for future lab-on-a-chip-based INP studies.

**Electronic supplementary material:**

The online version of this article (10.1007/s10404-018-2069-x) contains supplementary material, which is available to authorized users.

## Introduction

**Fig. 1:**
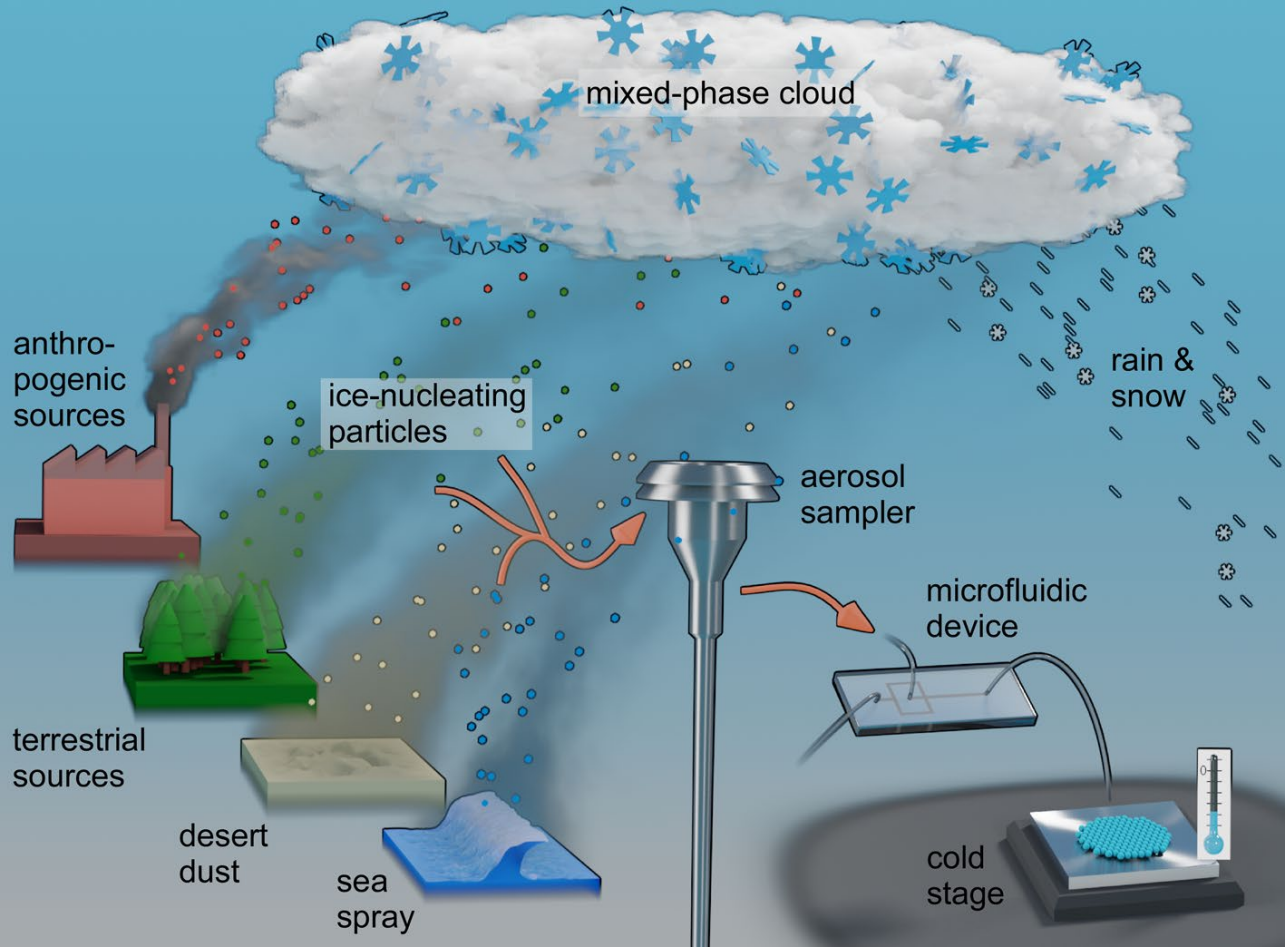
Schematic illustrating various sources of ice-nucleating particles (INPs) and the use of a microfluidic platform for quantifying INP concentrations. INPs can originate from anthropogenic, terrestrial, desert, and marine sources and can trigger ice formation in supercooled clouds that affects the cloud’s lifetime and radiative properties. We have developed a method of collecting aerosol particles onto filters in a way that is compatible for analysis with our microfluidic system. The aerosol sampler is designed to minimise losses of aerosol particles and has a cut-off at 10 µm, meaning it only samples aerosol below this size. Larger aerosol, rain droplets, or insects are removed via inertial impaction. We use polycarbonate track-etched filters which allow us to wash the particles off the filter into water with high efficiency for analysis with the microfluidic platform. Controlled cooling of the droplets until they freeze provides information on the INPs that can be used to test state-of-the-art global INP models

Clouds in the atmosphere affect Earth’s climate by reflecting and scattering solar radiation, as well as by reflecting, scattering, absorbing and emitting thermal radiation from the Earth’s surface, and as part of the hydrological cycle of evaporation and precipitation (Lohmann and Feichter 2005; Haywood and Boucher 2000). Clouds in the troposphere are able to supercool to temperatures below 0 °C in the absence of nucleation sites, remaining liquid down to temperatures below − 33 °C, whereupon they are prone to homogeneous freezing: the spontaneous freezing of water droplets (Pruppacher and Klett 1997). However, the presence of ice-nucleating particles (INPs) can trigger the formation of ice in supercooled clouds at much warmer temperatures via heterogeneous nucleation (Murray et al. 2012; Hoose and Möhler 2012), thus affecting the radiative properties of clouds (Vergara-Temprado et al. 2018). While atmospheric INPs are therefore clearly important, they are also incredibly rare, commonly comprising only around 1 in 10^3^–10^6^ ambient particles in the troposphere (Murray et al. 2012). They can originate from desert dust plumes (Tang et al. 2016; DeMott et al. 2003), marine sources (Burrows et al. 2013), and anthropogenic activities (Szyrmer and Zawadzki 1997), among others (Fig. 1), and their relative contributions can vary depending on time frame and location (Vergara-Temprado et al. 2017).

Despite these challenges, a range of instruments have been developed for the study of INPs (Vergara-Temprado et al. 2017; Hiranuma et al. 2015; DeMott et al. 2017; Wex et al. 2015). One of the more common types of instruments, alongside continuous flow diffusion chambers (CFDCs) and cloud expansion chambers, are cold stages that are used to explore immersion mode ice nucleation (Hiranuma et al. 2015). Here, particles immersed in droplets of water on a cold stage are cooled until they freeze, with the freezing temperatures providing information on the INPs present (Whale et al. 2015; Vali 1971; Tobo 2016; Budke and Koop 2015; Conen et al. 2011; Garcia et al. 2012; Stopelli et al. 2014; Knopf and Forrester 2011; Wright and Petters 2013; Fröhlich-Nowoisky et al. 2015; Gurganus et al. 2011; Vali and Stansbury 1966). However, a common issue with cold stage-based freezing experiments is that they often use relatively large droplet volumes (microlitre range). While this has the distinct advantage of having higher surface areas of nucleant per droplet and increased chances of containing rare INPs compared to smaller droplets, they will typically freeze at temperatures much higher than the homogeneous freezing point (e.g. > −25 °C) even in the absence of added INPs (Whale et al. 2015; Tobo 2016). The likely cause is that larger droplets have a greater chance of containing impurities or of contacting impurities on the supporting substrate (Vali 1971), with the consequence that the instrumental baseline is raised to the point that the ability to detect and quantify some INPs is limited.

With this in mind, some cold stages employ picolitre droplets that can be cooled to temperatures approaching homogeneous freezing, thereby greatly expanding the experimental temperature range. Such droplets are typically generated using a nebuliser (Koop et al. 1998; Atkinson et al. 2013; Knopf and Lopez 2009; Murray et al. 2011), a non-trivial technique that produces polydisperse droplets, or via emulsification with a vortex mixer (Hader et al. 2014; Wright et al. 2013) that also yields polydisperse populations. Microfluidic technology (Tarn and Pamme 2013; Whitesides 2006; Sackmann et al. 2014), on the other hand, enables the generation of very monodisperse droplets at high production rates for a range of applications (Casadevall i Solvas and de Mello 2011; Teh et al. 2008; Song et al. 2006; Chou et al. 2015; Zhu and Wang 2017). This is typically achieved via the injection of oil and water into a T-junction or flow-focussing junction channel design such that droplets of one phase are generated in the other (e.g. water-in-oil or oil-in-water droplets) as a result of the shear forces and interfacial tension at the oil–water interface. While microfluidic devices have been employed successfully in environmental monitoring (Marle and Greenway 2005; Jokerst et al. 2012; Campos and da Silva 2013), their application to the atmospheric sciences has been largely neglected. Only a handful of examples have alluded to atmospheric studies, but most have focused on either homogeneous freezing, the study of particles not typically found in the atmosphere (e.g. silver iodide), or ice-nucleating bacteria from a cryobiology viewpoint.

For example, Sgro et al. (2007) demonstrated the freezing of droplets containing cells in continuous flow for cryopreservation. Stan et al. (2009, 2011) developed a very elegant continuous flow platform featuring a finely controlled temperature gradient over which droplets were frozen. This was employed for the homogeneous freezing of water (Stan et al. 2009, 2011), including a study on the effect of electric fields on nucleation (Stan et al. 2011), and the heterogeneous freezing of water droplets containing silver iodide nanoparticles (Stan et al. 2009), a very effective INP used in artificial cloud-seeding (Marcolli et al. 2016).

While continuous flow freezing studies have been demonstrated, they require complex and careful preparation and operation to ensure accurate measurements in such a dynamic system. An alternative approach is to generate the droplets as normal before containing them in a static system that can be cooled. Sgro and Chu (2010) trapped water droplets in docking areas in a microfluidic channel that allowed the immiscible phase around the droplets to be exchanged, before cooling the entire device down in a custom-built, gas-cooled freezing chamber. Edd et al. (2009) trapped droplets of water, either pure or containing glycerol, in an array based on their “Dropspots” (Schmitz et al. 2009) device, before cooling the chip on a commercial cryomicroscopy stage. A similar platform was also recently employed by Reicher et al. (2018) for the analysis of desert dust-based INPs. Peckhaus et al. (2016) and Abdelmonem et al. (2017) used a commercial piezo-driven drop-on-demand generator to print an array of droplets on a cold stage for the study of mineral nucleators.

A straightforward method of monitoring droplets in a static system, without the need for complex microchannel geometries or spotting systems, is to simply collect the microfluidically generated droplets and dispense them directly onto a substrate on a cold stage. Riechers et al. (2013) employed this strategy for the study of homogeneous freezing using different-sized droplet populations via both a commercial cryomicroscopy stage and differential scanning calorimetry (DSC), with an in-depth analysis of the associated measurement errors. Weng et al. (2016) recently used a similar method for comparing the freezing points of water and heavy water (deuterium oxide) and included the first microfluidic study of Snomax^®^, a freeze-dried, non-viable preparation of the highly ice nucleation active (INA) bacteria, *Pseudomonas syringae*, from a cryobiology viewpoint.

However, there is a lack of microfluidic data for atmospherically relevant INPs. Further to this, although bio-aerosol sampling has been demonstrated via the use of microfluidic devices for the detection of aerosol chemical composition (Noblitt et al. 2009; Metcalf et al. 2016) and airborne pathogens (Jing et al. 2013, 2014; Ma et al. 2016; Bian et al. 2016; Choi et al. 2017; Jiang et al. 2016a, b), including on-site monitoring (e.g. in hospitals) (Noblitt et al. 2009; Jiang et al. 2016c; Liu et al. 2016), there is a notable lack of microfluidic applications for the study of the atmospheric sciences, in particular for the measurements of atmospheric INPs. Here, we employ the simple microfluidic strategy, described above, of combining microfluidic droplet generation and collection with a Peltier-based cold stage for the analysis of atmospheric INPs (Fig. 1), without the need for complex chip set-ups, microfabrication or spotting systems. In order to validate the platform, we performed measurements of known INPs present in desert dust (K-feldspar mineral particles) and as biological species [i.e. pollen- and fungal-based nanoscale INPs, and Snomax^®^ (*P. syringae*)]. In each case we then compared our results to those in the literature that have been obtained via a range of techniques. Finally, to demonstrate the applicability to atmospheric measurements, we performed preliminary experiments using the microfluidic platform for the analysis of atmospheric aerosol samples from two environments: (1) at a rural location in the UK and (2) during the UK’s annual Bonfire Night festival that involved the burning of bonfires and use of pyrotechnics.

## Experimental

### Chemicals and particles

Methanol, isopropanol, hydrofluoric acid (40%), and hydrochloric acid (37%) were purchased from Fisher Scientific (Loughborough, UK). Ammonium fluoride (40%), sodium bicarbonate, and Drierite desiccant were purchased from Sigma-Aldrich (Gillingham, UK). The fluorinated heat transfer oil, 3M™ Novec™ 7500 Engineered Fluid, was obtained from Fluorochem Ltd. (Hadfield, UK). The fluorinated surfactant, Pico-Surf™ 1 (5% w/w in Novec™ 7500 oil), was purchased from Dolomite Microfluidics (Royston, UK) and Sphere Fluidics Ltd. (Cambridge, UK) and further diluted to 2% w/w in Novec™ 7500 oil prior to experiments. Polydimethylsiloxane (PDMS, Dow Corning^®^ Sylgard^®^ 184 Kit) was obtained from Ellsworth Adhesives (East Kilbride, UK), while SU-8 2025 was purchased from MicroChem Corp. (Westborough, MA, USA).

Non-viable, lyophilised (i.e. freeze-dried) *P. syringae* bacterium was obtained as Snomax^®^ from York Snow, Inc. (York, PA, USA). Lyophilised cultures of *Fusarium avenaceum* fungus were sourced from the Centre for Agriculture and Biosciences International (CABI, Wallingford, UK). Wild silver birch pollen (*Betula pendula*, batch BETP.1310) was supplied by Pharmallerga (Lixov, Czech Republic). K-Feldspar (BCS 376 microcline) was sourced from the Bureau of Analysed Samples Ltd. (Middlesbrough, UK).

### Preparation of INP suspensions

All solutions and suspensions were prepared in purified water (18.2 MΩ cm at 25 °C, 0.22-µm-filtered) produced via a Milli-Q Academic Water Purification System (Millipore, Watford, UK), unless otherwise stated. 0.1% w/w suspensions of *P. syringae* and K-feldspar were prepared in purified water, with mixing achieved via mechanical agitation and vortexing.

Fungal (*F. avenaceum*) extract was prepared as described by O’Sullivan et al. (2016). Briefly, fungus was incubated for 3 days at 28 °C in potato dextrose broth (PDB), and then a portion of the mycelium was separated and resuspended in 50 mL of purified water (0.02 g mL^−1^). The suspension was agitated by hand and filtered through 0.2-µm cellulose acetate filters, then diluted 1/20 in purified water. Four millilitres of this suspension was diluted to 10 mL with purified water and then stored in a freezer (− 20 °C, approximately 18 months) until ready for use.

Birch pollen (*B. pendula*) extract was prepared as described by O’Sullivan et al. (2015). Briefly, 1 g of dried pollen was suspended in 50 mL water (i.e. 2% w/v), shaken, and then stored in a fridge for 12 h where it was allowed to settle. The pollen solution was subsequently shaken, then filtered through an 11-µm nylon net filter and a 0.2-µm cellulose acetate filter to yield the final suspension of pollen extract, which was stored in a freezer (− 20 °C) until ready for use.

All INP suspensions were mechanically agitated and then vortexed for 1 min immediately prior to use in order to ensure homogeneous distribution of the particles, before they were drawn into a syringe for injection into the microfluidic device.

### Atmospheric sampling and preparation of suspensions

Sampling of atmospheric aerosol particles is notoriously challenging, with the design of the sampling system playing an important role in the minimisation of particle losses; smaller particles are prone to diffusional losses, while larger particles can be lost by impaction against the tube walls at bends in the pipework (Brockmann 1993). In addition, if the air flow speed deviates from the velocity of the air relative to the inlet, aerosol concentrations can be either enhanced or reduced due to sub-isokinetic or super-isokinetic sampling. Hence, care is needed to ensure that particles are sampled representatively via appropriate design of a sampling head and flow tube system, thereby limiting sampling errors. INPs commonly have sizes in the *coarse mode* (radii, *r* > 1 µm) or even *accumulation mode* (0.1 ≤ *r* ≥ 1 µm), as opposed to the *nuclei mode* (*r* < 0.1 µm) (Pruppacher and Klett 1997), meaning that systems capable of sampling PM_10_ (particulate matter with diameters ≤ 10 µm) are required. It is therefore very important in sampling atmospheric INPs that losses in the aerosol sampling system and subsequent handling are minimised. In this particular case, it was also necessary to employ a sampling strategy that was compatible with a microfluidic platform by providing a means of obtaining an aqueous suspension of the sampled particles.

We opted to use filter-based platforms with United States Environmental Protection Agency (EPA) standard PM_10_ sampling heads and downtubes to collect INP, whereby a membrane filter is positioned in a flow tube as air is pulled through it, thus depositing particles directly onto the filter for subsequent analysis with the microfluidic system. This strategy was employed in two field campaigns: (1) at a rural location and (2) during the UK’s annual Bonfire Night festival.

The campaign at the rural location, which is described in more detail elsewhere (O’Sullivan et al. 2018), took place at the University of Leeds Field Research Unit (UK) from September to October 2016 and included the first deployment of our mobile laboratory for ice nucleation research, the “IcePod”. The IcePod was fitted with a custom-built external air sampling inlet and downtube, each suitable for PM_10_ collection, that fed into the mobile laboratory. Isokinetic sub-sampling from the primary flow allowed us to simultaneously sample into two particle sizing instruments and two Savillex filter holders (QMX Laboratories, Thaxted, UK). The filter holders housed Whatman^®^ Nucleopore™ Track-Etched Membrane polycarbonate filters (47 mm diameter, 0.4 µm pore size, Sigma-Aldrich, UK) that were connected to pumps via mass flow controllers (MFCs), allowing air to be pulled through the filters at 5 L min^−1^ for the collection of aerosol particles. The particle sizing instruments consisted of an Aerodynamic Particle Sizer (APS) Spectrometer 3321 (TSI Inc., Aachen, Germany), which measured particle sizes from 0.5 to 20 µm, and a Scanning Mobility Particle Sizer (SMPS) Spectrometer 3938 (TSI Inc.), which measured particles from 1 to 1000 nm.

The Bonfire Night campaign, which is also described in further detail elsewhere (Adams et al. 2018), took place on 5 November 2016 at the University of Leeds (UK). A commercial, portable sampling unit was employed for the collection of aerosol particles onto Whatman^®^ Nucleopore™ polycarbonate filters at 16.7 L min^−1^. The sampling unit was a BGI PQ100 Air Sampling System with a PM_10_ inlet and downtube (shown schematically in Fig. 1, Mesa Laboratories, Inc., Butler, NJ, USA) that was designed to EPA requirements and is used as an EPA Federal Reference Method for PM_10_ (Designation no. RFPS-1298-124). Aerosol samples were collected over the course of 1 h, with samples taken at a rate of 1 per hour for 8 h, with 15 min changeover time. An APS, SMPS, and black carbon monitor were also used to monitor ambient particle concentrations.

Having obtained filters with aerosol particles collected onto them, from either campaign, the next step was to suspend the particles in water for introduction into the microfluidic platform. We therefore adopted a method based on that of Hill et al. (2014) in which adsorbed particles were washed off the filters and into suspension with high efficiency. Briefly, the polycarbonate filters were carefully inserted, using tweezers, into 50-mL centrifuge tubes (Sarstedt Ltd., Leicester, UK), and then 5 mL of water added. The tube was shaken vigorously and left on a rotary mixer (Clifton^®^ RM-1, Nickel-Electro Ltd., Weston-super-Mare, UK) at 30 rpm for 1 h to wash the particles off the filter and into the water. Samples were frozen (− 20 °C) until ready for use, whereupon they were thawed, shaken vigorously, left on a rotator for 1 h and vortexed before being drawn into a syringe for injection into the microfluidic device. The same samples were also analysed using the microlitre Nucleation by Immersed Particle Instrument (µL-NIPI) (Whale et al. 2015), a cold stage immersion mode freezing instrument in which ~ 50 droplets (1 µL volume) are pipetted onto a Stirling engine based cold stage (EF600, Grant-Asymptote Ltd., Cambridge, UK) and then cooled down.

### Microfluidic chip fabrication and set-up

**Fig. 2:**
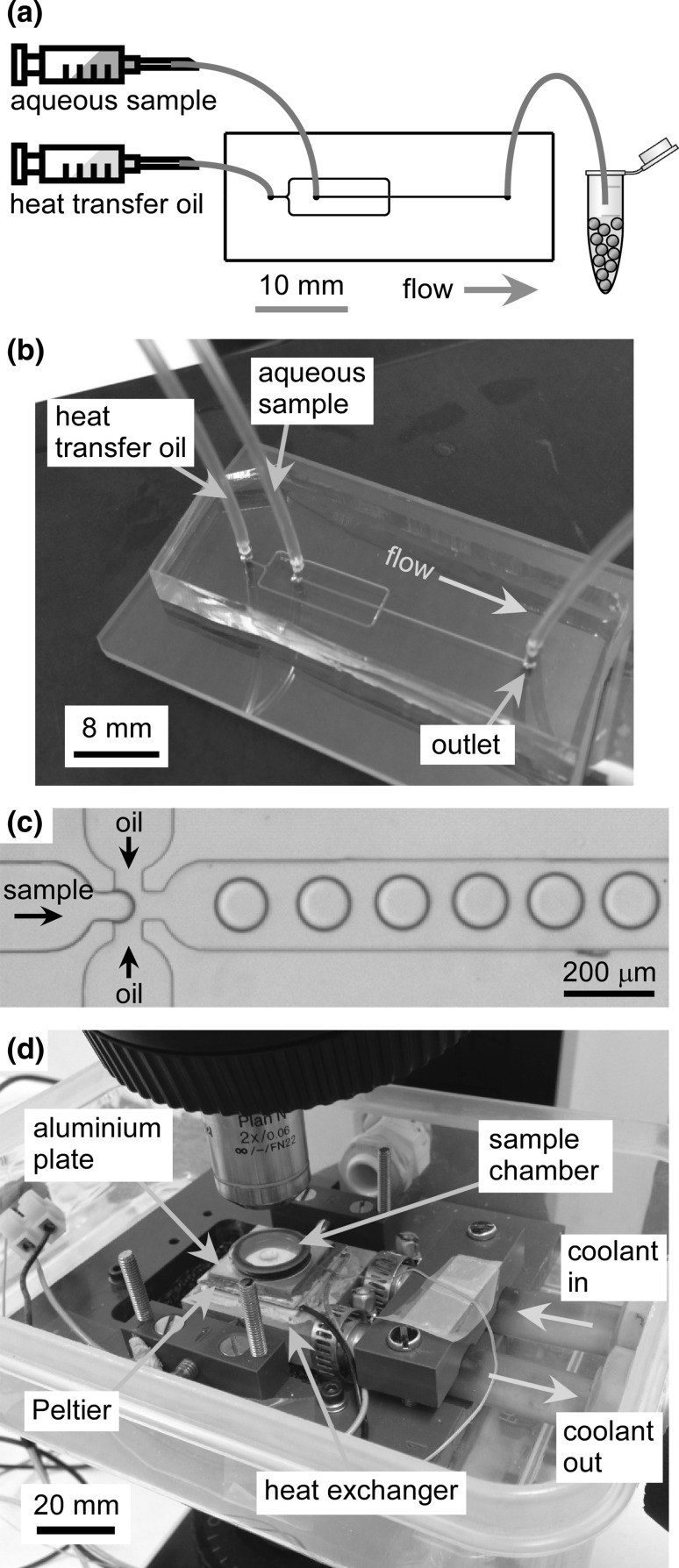
Overview of the apparatus and procedure for the freezing of microfluidically generated droplets containing ice-nucleating particles (INPs). **a** Chip design and operating principle for the generation and collection of aqueous droplets, containing INPs or sampled atmospheric aerosol, in a fluorinated heat transfer oil. The scale bar refers only to the chip design. **b** Microfluidic device fabricated out of polydimethylsiloxane (PDMS), **c** generation of water-in-oil droplets in the microfluidic device, **d** collected water-in-oil droplets were pipetted into a sample chamber and placed onto a Peltier-based cold stage for freezing experiments

The microfluidic chip design consisted of a flow-focussing (Anna et al. 2003) droplet generation junction (Fig. 2a) that was designed in CleWin 5.2 Layout Editor software (WieWeb Software, Hengelo, The Netherlands). The channels had a width of 200 µm, apart from the nozzle area that featured a width of 60 µm, and the main channel downstream of the flow-focussing junction was 12.5 mm long. Devices were fabricated out of polydimethylsiloxane (PDMS) (Fig. 2b) using standard rapid prototyping and soft lithography procedures and featured 55-µm-deep microchannels (McDonald et al. 2000; Duffy et al. 1998). Briefly, a master was prepared by spin-coating SU-8 2025 negative photoresist onto a silicon wafer (PI-KEM, Tamworth, UK) and exposing the channel design onto it via a direct laser writer (MicroWriter ML, Durham Magneto Optics Ltd., Durham, UK). Following photodevelopment to reveal the channel structure on the silicon wafer, PDMS was poured over the master, degassed, and then cured at 75 °C for 1 h before being peeled off the master. Finally, 1-mm-diameter access holes were punched into the PDMS devices, which were then bonded to glass microscope slides after treatment with oxygen plasma (Zepto Version B, Diener Electronic GmbH, Germany).

Polyethylene tubing (Smiths Medical 0.38 mm i.d. × 1.09 mm o.d., tubing, Harvard Apparatus (Biochrom Ltd.), Cambridge, UK) was inserted into the continuous phase inlet, the dispersed phase inlet, and the outlet. The continuous phase inlet tubing was interfaced to a glass syringe (250 or 500 µL, SGE, Sigma-Aldrich, UK) via a low dead volume connector (Kinesis, St. Neots, UK). In order to avoid contamination between different INP suspensions, the dispersed phase inlet tubing was interfaced to disposable plastic syringes (1 mL, Henke-Sass Wolf, VWR, Lutterworth, UK) via syringe needles. The syringes were each inserted into syringe pumps (PHD Ultra, Harvard Apparatus, UK) for the introduction of the continuous phase and dispersed phase into the chip. The continuous phase, containing 2% w/w Pico-Surf™ 1 fluorinated surfactant in Novec™ 7500 fluorinated oil, was pumped into the device at a flow rate of 20 µL min^−1^. The dispersed phase consisted of either water or INP suspension that was vortexed for 30 s immediately before being drawn into the syringe, placed on the syringe pump and pumped into the chip at a flow rate of 16 µL min^−1^. Monitoring of droplet formation (Fig. 2c) in the microchannel was achieved via a microscope (Leica DMLM, Milton Keynes, UK). The end of the outlet tubing was inserted into an Eppendorf microcentrifuge tube (1.5 mL, Fisher Scientific, UK) for the collection of water-in-oil droplets from the microfluidic device.

### Fabrication of microwells in cover slips

In order to perform freezing experiments on collected droplets, microwells were fabricated in glass cover slips to ensure that the droplets remained in the field of view rather than sliding out of position. Circular siliconised glass cover slides (22 mm diameter, 0.22 mm thick, Hampton Research, Aliso Viejo, CA, USA) were coated with masking tape (INT 600 Masking Tape, Intertronics, Kidlington, UK), and then a small section (~ 5 mm^2^) was cut out of the centre of the tape using a scalpel or tissue biopsy punch to reveal the glass surface (Fig. S1 in the ESI). The masked cover slips were immersed in a glass etching solution (containing 1% hydrofluoric acid (HF), 5% ammonium fluoride, 10% hydrochloric acid, 84% water) and etched to a depth of 30 µm. The cover slips were then removed from the etching solution, neutralised in saturated sodium bicarbonate solution, and washed with purified water. Finally, the masking tape was removed to reveal a 30-µm-deep microwell (~ 5 mm^2^) in the centre of each of the cover slips. Lids for the etched cover slips were prepared by taking an unetched cover slip and gluing a nitrile rubber O-ring (16.0 mm i.d., 21.2 mm o.d., 2.6 mm thick, RS Components, Corby, UK) to it with Araldite epoxy resin (RS Components, UK).

### Peltier-based cryomicroscopy stage

In order to perform freezing experiments of the collected water-in-oil droplets, a Peltier-based cooling stage (Fig. 2d) was designed and built in-house. A 30 × 30 × 3.6 mm^3^ Peltier element module (ET-127-10-13, 37.9 W, 3.9 A, 15.7 V, RS Components, UK) was mounted onto a liquid heat exchanger (39 × 39 × 12 mm^3^, fabricated out of aluminium) with thermal paste (RS Components, UK). The Peltier module and heat exchanger were housed within a sealable plastic box, providing an airtight chamber that was mounted onto a microscope (BX51, Olympus, Southend-on-Sea, UK) having a 2 × objective and a reflected light module. Images were captured via a Microsoft LifeCam HD web camera that had been modified to enable attachment to the microscope via a C-mount. The liquid heat exchanger was held in position via three screws, each sharpened to a point, that were fixed to the sealable plastic box. Manipulation of the screws allowed positioning of the cooling stage under the microscope objective, while the small contact area of the sharpened screws minimised the thermal mass on the heat exchanger. The lid of the plastic box had a hole drilled through it for the microscope objective to pass through, and a small Perspex shield placed around the objective was used to seal it against the lid via O-rings and vacuum grease, thereby forming a sealed cold chamber (Fig. S2 in the ESI). Prior to sealing the chamber, small pots of Drierite desiccant were placed inside in order to remove moisture and so prevent condensation and frosting upon cooling.

An aluminium plate (25 × 25 × 2 mm^3^) that had been polished to a reflective finish was fixed to the top of the Peltier element using thermal paste (Arctic Silver 5, Amazon, UK), acting as the platform upon which the sample was placed. The plate featured a drill hole (0.8 mm diameter) into which a thermocouple (0.13 mm diameter, 5SC series K-type, Omega Engineering Ltd., Manchester, UK), coated in thermal paste, was inserted for temperature measurements. Polymer tubing (9 mm i.d., 12 mm o.d.) was connected to the liquid heat exchanger through the side of the plastic sandwich box and interfaced to a recirculating chiller (KTC Chiller, Applied Thermal Control, Whitwick, UK) set to − 8 °C. The chiller pumped Hexid Heat Transfer Fluid (Applied Thermal Control, UK) through the liquid heat exchanger in order to remove the heat generated by the underside of the Peltier module during its operation, allowing the Peltier cooling stage to achieve a minimum temperature of − 47 °C.

The Peltier module and thermocouple were interfaced to a custom-built proportional–integral–derivative (PID) controller based on an Arduino Nano (RS Components, UK) microcontroller, allowing feedback-based temperature control via a program written in Python software (Python Software Foundation, Delaware, USA). The program also controlled the web camera, allowing the synchronised collection of capture images and temperature measurements at a rate of 1 s^−1^.

### Freezing experiments

Microwell cover slips were first washed with methanol, isopropanol, and purified water prior to use. An aliquot (2 µL) of the water-in-oil droplet suspension collected from the microfluidic device was pipetted into the microwell of an etched glass cover slip with an additional 2 µL of oil added to ensure the droplets remained suspended. (The heat transfer oil is prone to evaporation, which can lead to droplets becoming pressed together and affecting their freezing characteristics if the oil is allowed to dry out.) A lid consisting of an O-ring and a cover slip was immediately placed onto the microwell cover slip and sealed with vacuum grease, forming a small sample chamber that prevented evaporation of the sample. The sample chamber was placed onto the polished aluminium plate of the Peltier-based cooling stage (Fig. 2d), and pots containing Drierite desiccant were added to the cold chamber, after which the cold chamber was sealed (Fig. S2 in the ESI) and cooling was initiated. The stage was cooled rapidly (at 10 °C min^−1^) until it was within 10 °C of the expected initial freezing events of the sample, after which it was cooled more slowly (at 1 °C min^−1^) for observation of the droplet freezing events. Droplet diameters in the sample chamber were analysed from the captured images using ImageJ software (https://imagej.nih.gov/ij/), with a measurement resolution of 3 µm pixel^−1^ given the microscope objective used. Analysis of freezing events was performed using a program written in Python that synchronised the temperature logs and recorded images, allowing the user to scan through the images and record the temperature at which each event occurred.

### Temperature calibration

A platinum resistance thermometer (PRT) probe (Model 5608, ± 0.0013 °C, Fluke Corporation, USA) and digital readout stack unit (Model 1560, Fluke Corporation, USA) were calibrated by the National Physical Laboratory (NPL, Teddington, UK). This probe was then used to calibrate a high gauge, fast response PRT probe (Model 5622-05 Plamic Pt 100 Ω, ± 0.04 °C, Fluke Corporation, USA) with a data logger (Model 1524, ± 0.01 °C, Fluke Corporation, USA), in addition to a thermocouple (0.13 mm diameter, 5SC series K-type, ± 1.1 °C, Omega Engineering Ltd., UK) with a data logger (TC-08, ± 0.025 °C, Pico Technology, St. Neots, UK). The thermocouple inside the polished aluminium plate on the Peltier-based cooling stage was calibrated against the fast response PRT probe (Model 5622-05) by placing it inside the aluminium plate alongside the thermocouple and cooling the stage to − 45 °C.

While the above allowed determination of the temperature of the aluminium plate, it did not provide an indication of the temperature experienced by the water-in-oil droplets inside the sample chamber. Therefore, an effort was made to estimate the temperature offset between the droplets inside the chamber and the thermocouple, since the droplets were separated from the aluminium plate measurement point by 0.5-mm-thick aluminium and a 0.22-mm-thick glass cover slip. Since the freezing experiments were performed at a constant cooling rate, a particular concern was that the offset could increase during the cooling process; hence we sought to characterise and correct for such a “lag”.

A calibration chamber was prepared that consisted of two glass circle cover slips supported by two nitrile rubber O-rings, all of which were superglued together. One of the O-rings featured a small cutaway through which the chamber was filled with Novec™ 7500 oil, prior to insertion of the calibrated thermocouple (Omega 5SC series probe with a Pico Technology TC-08 data logger) and sealing of the cutaway with vacuum grease. The oil-filled calibration chamber was placed on top of the polished aluminium plate, and great care was taken to make sure the thermocouple was located on the surface of the lower glass cover slip and that it was surrounded by oil. This ensured that measurements using the thermocouple were taken in a similar oil-on-cover slip environment as for the droplets. However, while the normal sample chamber would have a headspace of air above the water-in-oil droplet suspension, fully encompassing the thermocouple in oil (including several millimetres of length of the thermocouple wires) inside the calibration chamber ensured that temperature effects caused by the thermocouple itself (e.g. conduction of heat along the length of the thermocouple from the ambient air outside the calibration chamber) would be minimised.

Thermocouple measurements were taken inside the oil-filled calibration chamber at a number of Peltier stage set points down to − 45 °C, while continuous measurements were also taken at a cooling rate of 1 °C min^−1^ down to − 45 °C in order to determine the extent of any lag in cooling between the aluminium plate and oil. The offset between the plate temperature and the thermocouple temperature increased as the stage was cooled down, from + 0.04 °C when the aluminium plate was at 0 °C, to a difference of + 0.96 °C when the plate was at − 40 °C during the 1 °C min^−1^ ramp. This represented a worst-case scenario in terms of offset. However, while the offset was not constant, its linearity (*R*^2^ = 1) allowed a correction factor to be incorporated into the measured temperatures of the droplet freezing events during data analysis. Although the uncertainty of the thermocouple was quoted by the manufacturer as being ± 1.1 °C, the various calibration tests we performed had suggested that the precision of the thermocouples was lower than the quoted value, while demonstrating high accuracy (following the application of appropriate correction factors) when used alongside the PRT probes. With this in mind, we estimated a temperature uncertainty of ± 0.5 °C in the final measurements.

## Results and discussion

### Microfluidic droplet generation

The choice of oil and surfactant combination for droplet production was important during freezing experiments since the droplets must remain stable in sub-zero temperatures, in particular down to around − 40 °C to cover the relevant range, while high thermal conductivity was also required to enable rapid, controlled cooling. This somewhat limited the options compared to room temperature experiments, and Hauptmann et al. (2016) summarise the various oil systems that have been employed in the literature for the freezing of aqueous droplets.

Novec™ 7500 fluorinated oil is designed and sold as a heat transfer fluid, thus being ideal for cooling aqueous droplets suspended in the oil with minimal temperature difference, while its lowest working temperature is − 100 °C.^1^ Perfluoropolyether–polyethylene glycol (PFPE–PEG) amphiphilic block copolymer is a fluorinated surfactant that was developed to provide stable and bio-compatible water-in-fluorinated oil droplets (Holtze et al. 2008), and which is available commercially as Pico-Surf™ 1 from Dolomite Microfluidics^2^ and Sphere Fluidics,^3^ or as EA surfactant from RainDance Technologies, while it can also be “home-made” from Krytox^®^ PFPE grease (DuPont) (Holtze et al. 2008; Chen et al. 2011; Cho 2013; Shim et al. 2013). The combination of Novec™ 7500 oil (or other fluorinated oils such as FC-40) and PFPE–PEG fluorinated surfactant has been shown to produce monodisperse and extremely stable water-in-oil droplets in microfluidic devices (Holtze et al. 2008; Cho 2013; Shim et al. 2013; Mazutis et al. 2009, 2013; Baret 2012; Abate et al. 2010; Joensson et al. 2011; Baret et al. 2009), with the droplets able to assemble into close-packed arrays without coalescence despite effectively touching each other, even after storage over several days (Holtze et al. 2008; Cho 2013). The PFPE–PEG surfactant allows a thin film (on the order of 10 nm) of fluorinated oil to exist between the droplets that helps to stabilise them, while the interfacial tension has been estimated at around 3 mN m^−1^ (Holtze et al. 2008). Furthermore, fluorinated oils are both hydrophobic *and* lipophobic/oleophobic; hence, they tend to reject non-fluorinated species whether they are hydrophilic or hydrophobic, which was an important property here for ensuring that the various INP species were unable to partition into the oil phase. Other attractive properties of the Novec™ 7500 oil include its low toxicity and non-flammability, and it is exempt from the EPA’s definition of a volatile organic compound (VOC) as it does not contribute to photochemical smog, having been developed as a non-ozone-depleting, low-global warming potential (GWP) alternative to other perfluorocarbon (PFC) and PFPE heat transfer fluids (see footnote 1).

As a result of these properties, this oil/surfactant system has seen success in microfluidic applications where rapid heat transfer is applied to a monolayer of close-packed water-in-oil droplets, e.g. thermocycling for polymerase chain reaction (PCR) amplification of DNA (Schuler et al. 2016; Zhang et al. 2016; Rhee et al. 2016; Pekin et al. 2011) and freezing in cryobiology studies (Weng et al. 2016). Therefore, the Novec 7500™ oil and PFPE–PEG surfactant (specifically Pico-Surf 1™ from Dolomite Microfluidics and Sphere Fluidics) system was also employed here as the microfluidic continuous phase (CP). The dispersed phase (DP), consisting of either water only or aqueous samples of INPs, was pumped into the PDMS microfluidic device at 16 µL min^−1^, alongside the CP at 20 µL min^−1^. The high flow rates allowed the rapid production of water-in-oil droplets (Fig. 2c), thereby reducing the time available for some of the denser INPs to settle out of suspension. The time taken from drawing the INP suspension into the syringe and tubing to placing it onto the syringe pump, connecting the tubing to the chip, and generating a population of droplets was approximately 3 min. The droplets produced had average diameters of 83–99 µm (measurement resolution = 3 µm pixel^−1^) depending on the sample; further details for each sample are given in the following sections and in Fig. S3 of the ESI. While the generated droplets were very stable upon their generation due to the fluorinated oil and surfactant combination used, it was found that after freezing the droplets their stability broke down after thawing, with the melted droplets coalescing to form much larger droplets. This may be due to deterioration of the surfactant or its expulsion from the water droplets upon freezing, but effectively means that performing freeze–thaw experiments with this oil and surfactant combination is not a viable option, limiting the system instead to one-off freezing experiments with a given aliquot of droplet suspension.

Freezing of the droplets was performed by dispensing an aliquot (2 µL) of the collected droplet suspension onto a glass cover slip with a microwell etched into it. The density of water is lower than that of Novec™ 7500 oil, resulting in the aqueous droplets floating on the thin layer of heat transfer oil. This meant that the droplets were prone to floating out of the field of view on the microscope when using a normal, flat cover slip, while using the HF-etched shallow microwells meant that the droplets could be contained within the viewing area. Flat cover slips actually worked perfectly well for freezing experiments provided care was taken with droplet placement and subsequent handling, but we found the etched slips to be more reliable. In lieu of HF etching, we also found that microscope cavity slides performed a similar and satisfactory function, although their thickness means that careful temperature calibration would be required. An easier method could be to use wide, flat, rectangular cross section capillary tubes with thin walls (e.g. VitroTubes™ from VitroCom) into which a population of droplets can be drawn by capillary action, as has been demonstrated successfully for a variety of applications requiring monolayers of stationary, close-packed droplets (Mazutis et al. 2013).

A further consequence of the droplets floating on the thin oil layer is that the droplets would not contact the glass cover slips that they were deposited on. Many immersion mode cold stage freezing techniques involve the dispensing of aqueous droplets onto glass slides, but the slides must be hydrophobic in order to prevent the droplets from spreading on the slide and potentially freezing at higher temperatures, a particular issue for the homogeneous freezing of pure water droplets. However, in our method the surface properties of the slides are not important since the droplets do not actually contact the surface. This also provides the advantage that the microwell glass slides can be thoroughly cleaned between each run and then reused for freezing experiments, since there is little to no risk of contaminating the oil-encompassed droplets. Repeated use of the glass microwell slides showed no effect on the freezing properties of pure water, while tap water samples and aerosol samples also showed some droplets freezing homogeneously on the reused slides. Thus, while etching of the glass slides with hazardous HF is an additional step to the overall procedure, a single batch of etched slides can be used many times if handled carefully.

### Analysis of atmospherically relevant INPs

**Fig. 3:**
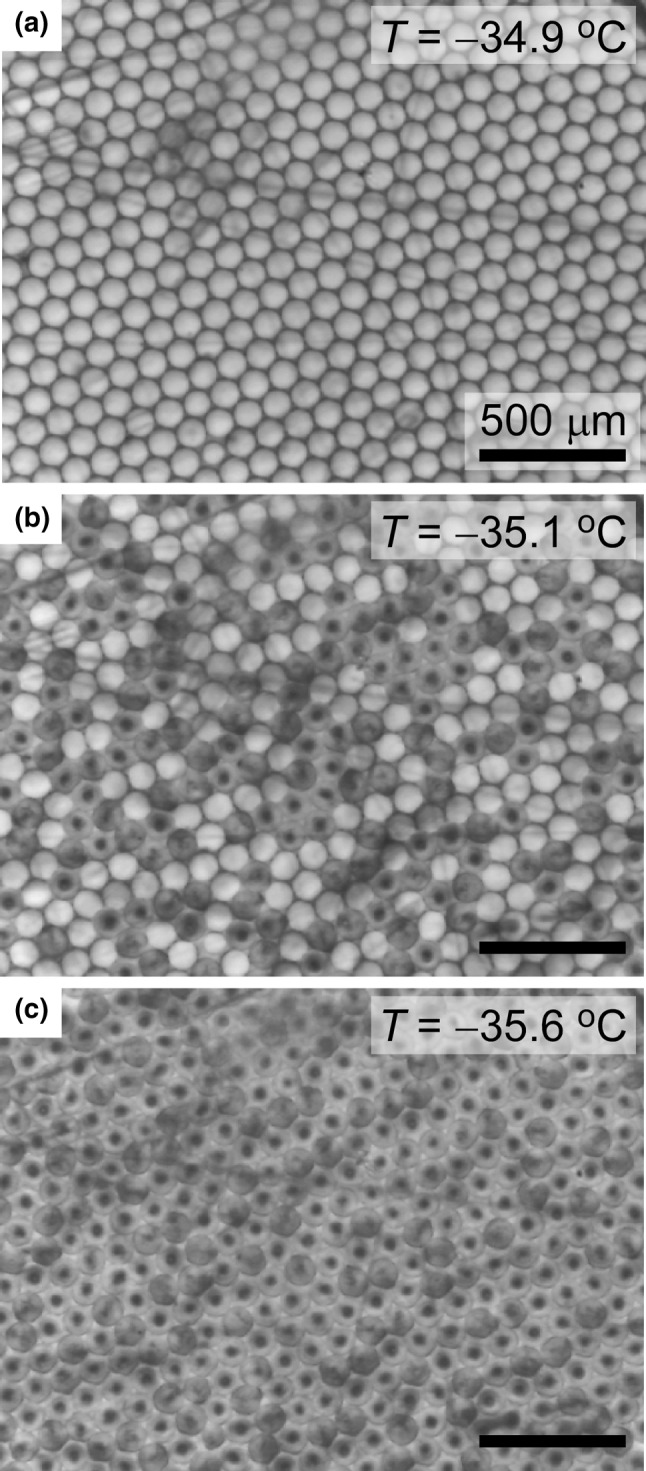
Photographs showing the freezing of pure water droplets that have been pipetted into the sample chamber on the microscope cold stage. Liquid droplets were clear and colourless, but when they froze they became darker and exhibited a black spot where nucleation occurred. **a** Before freezing, **b** with half of the droplets frozen (i.e. *T*_50_), and **c** with all of the droplets frozen. The freezing temperatures were recorded for each droplet and used to calculate fraction frozen (*f*_ice_(*T*)) curves. The scale bar in (**a**) applies to all three photographs

Droplets generated via the microfluidic device were collected and then cooled via the Peltier-based cooling stage (Fig. 3). Upon freezing, the droplets changed from being clear and colourless to grey with a black nucleus in the middle, making it easy to distinguish freezing events. Images and temperature measurements were taken every second, from which the temperatures at which each aqueous droplet froze could be determined with the help of a Python program written in-house. 250–500 droplets were analysed per sample, depending on the number of droplets that were in the frame during the particular set of experiments. The Python program did not allow automated analysis, but did help to greatly reduce the time needed to manually record the temperatures at which droplet freezing events occurred, taking around 30–40 min of analysis per experiment. Figure 3 shows an example of the image frame being filled with droplets and highlights the maximum number of droplets that could be analysed per experiment based on the optics used, a number that could be increased if needed by either generating smaller droplets, using lower-magnification optics, or by using a CCD camera with a wider field of view. The fraction of droplets frozen, *f*_ice_(*T*), by temperature *T* for a range of atmospherically relevant INP was calculated (Vali 1971, 1994; Connolly et al. 2009; Niedermeier et al. 2010; Niemand et al. 2012):

1 $$f_{\text{ice}} \left( T \right) = \frac{{n_{\text{ice}} \left( T \right)}}{{n_{\text{tot}} }},$$

where *n*_ice_(*T*) is the total number of frozen droplets at temperature *T* and *n*_tot_ is the total number of droplets. In order to avoid contact freezing, i.e. the triggering of a nucleation event in one droplet by the freezing of an adjacent droplet, it was important to avoid evaporation of the fluorinated oil that the droplets were suspended in; if too much evaporation occurred the droplets would become “squashed” together, forming a honeycomb-like structure that would freeze in sections of droplets rather than on a droplet-by-droplet basis. However, by pipetting only a small population of droplets onto the sample chamber slide (2 µL) and adding a further 2 µL of oil before sealing the sample chamber with the lid, excessive evaporation of the oil and hence the freezing of neighbouring droplets could be avoided.

**Fig. 4:**
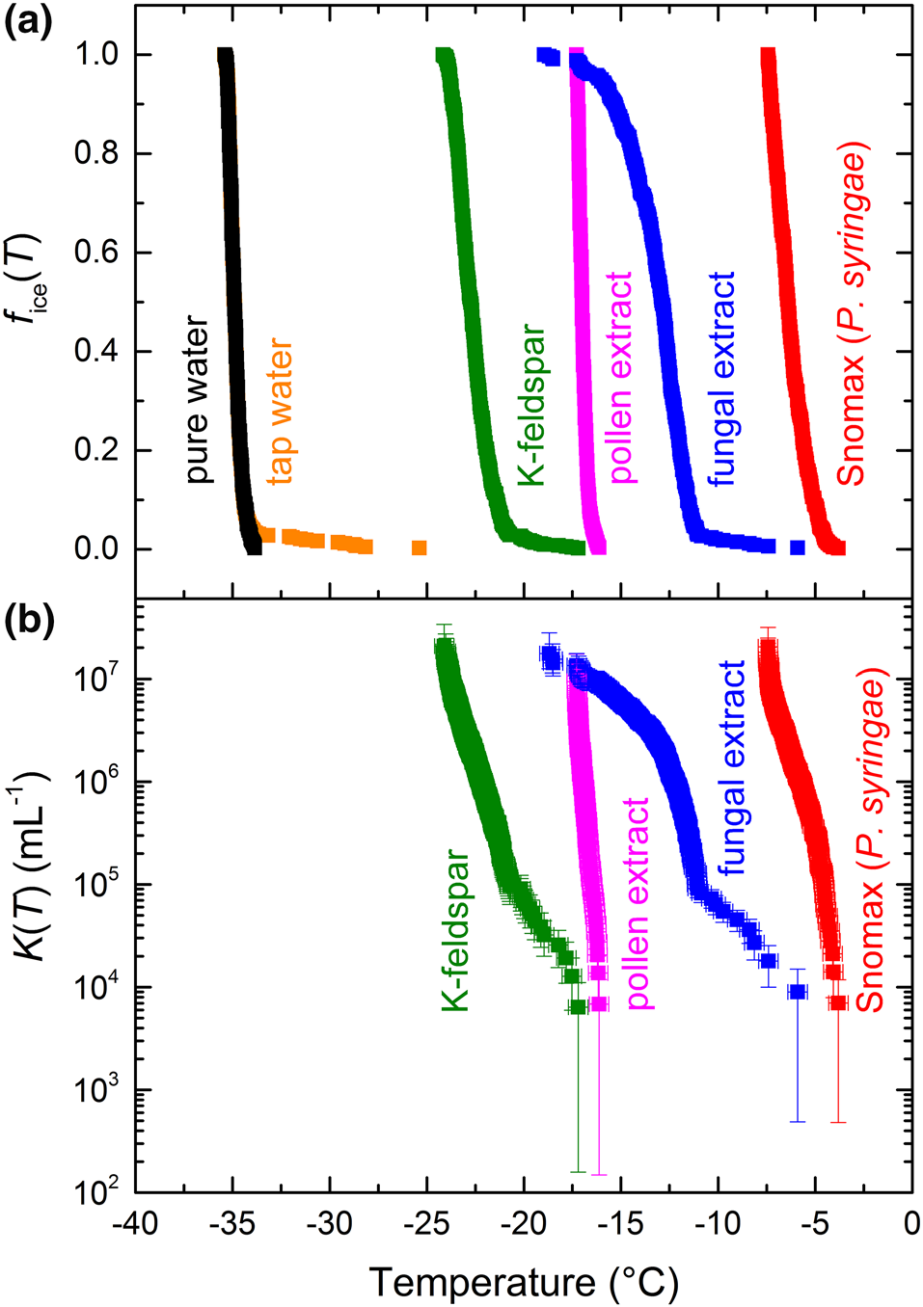
Ice nucleation studies for water and a range of atmospherically relevant INPs of dust and biological origins. **a** Fraction frozen (*f*_ice_(*T*)) curves, **b** the cumulative number of ice nucleation sites per millilitre (*K*(*T*)) for the various INPs. The temperature uncertainty was estimated to be ± 0.5 °C. The errors in *K*(*T*) were calculated from the variance in droplet size and from estimates of the Poisson counting errors obtained by performing Monte Carlo simulations following the method shown by Harrison et al. (2016)

Droplets containing purified water without the addition of INPs were used as a blank. The fraction frozen (*f*_ice_(*T*)) curves are shown in Fig. 4a, including correction for the temperature lag between the aluminium plate and the sample chamber. The *f*_ice_(*T*) results demonstrate the range over which different atmospheric INPs trigger the freezing of water, from the highly active *P. syringae* bacteria (in the form of Snomax^®^) at − 3.8 to − 7.4 °C, to K-feldspar mineral dust at − 17.2 to − 24.2 °C. Purified water, in the absence of any added INPs, was shown to freeze homogeneously between − 33.9 and − 35.4 °C. A sample of tap water was analysed to demonstrate the difference between a sample of pure water and a “contaminated” water sample, with the presence of INPs in the tap water triggering ice formation at temperatures as high as − 25.4 °C. However, the amount of INPs in the tap water sample was actually quite low, with only a small fraction (~ 3%) of the droplets freezing at temperatures higher than the pure water sample (the remainder of the tap water data points are obscured by the pure water data points in Fig. 4a since they displayed almost identical freezing characteristics below about − 33.9 °C).

Interestingly, the droplets generated from the tap water sample appeared to be more prone to triggering each other when freezing. This was possibly due to the presence of fluoride ions or other contaminants in the tap water allowing interactions between droplets even across the fluorinated oil and surfactant interfaces. However, this triggering effect could largely be avoided by ensuring extra care was taken to reduce the evaporation of the oil, thereby preventing the droplets from becoming too squashed together. The Bonfire Night samples also suffered a little from this triggering effect, but to a lesser extent than the tap water, and again this could be largely prevented by taking more care during preparation of the freezing experiment.

Assuming a singular approximation, in which ice nucleation is considered to be a temperature-dependent and time-independent process, each droplet containing INPs will freeze at a characteristic temperature that will depend upon the nature of the INPs. According to the singular model, the cumulative number of ice nucleation sites per unit volume of water, *K*(*T*), on cooling to temperature *T* can be calculated from the fraction frozen curves according to (2), where *V* is the volume of a droplet and *f*_water_ is the fraction of droplets that remain liquid by temperature *T* (Murray et al. 2012; Vali 1971; Budke and Koop 2015):

2 $$K\left( T \right) = \frac{{ - \ln \left( {1 - f_{\text{ice}} \left( T \right)} \right)}}{V} = \frac{{ - \ln \left( {f_{\text{water}} \left( T \right)} \right)}}{V},$$

The results for *K*(*T*) (per mL) of the various samples are shown in Fig. 4b, with error values calculated based on the variation in droplet size and on the Poisson counting errors determined via Monte Carlo simulations as performed previously in Harrison et al. (2016). It should be noted that there is no plot for pure water since, if water freezes homogeneously, there are no active sites present and hence no *K*(*T*). No plot for tap water is shown as that was simply tested for comparison to pure water. From *K*(*T*), a number of INP properties can be determined regarding the number of active sites on the particles between 0 °C and temperature *T*, based on the mass concentration, *C*_m_, of INPs:

3 $$\frac{K\left( T \right)}{{C_{\text{m}} }} = n_{\text{m}} \left( T \right) = n_{\text{s}} \left( T \right) \cdot S = n_{\text{n}} \left( T \right) \cdot N,$$

where *n*_m_(*T*) is the active site density per unit mass of INPs, *n*_s_(*T*) is the number of active sites per surface area, i.e. the ice-active surface density, *S* is the surface area of INPs per droplet, *n*_n_(*T*) is the active site density per particle number, and *N* is the specific particle number, i.e. the number of particles per sample mass. These various active site density values provide a standard by which measurements of a material's ice nucleation efficiency can be compared between instruments, as demonstrated in the literature (Hiranuma et al. 2015; Wex et al. 2015). The median freezing temperature, *T*_50_, i.e. the temperature at which 50% of the droplets have frozen, is also sometimes used for comparison, although care needs to be taken when quoting and interpreting this value since *T*_50_ can depend greatly on droplet size, INP concentration, and cooling rate. The *T*_50_ values for the samples analysed here are shown in Fig. S4 in the ESI.

Having established the fraction frozen curves and INP concentrations for the various samples, each was then further investigated in terms of its ice-nucleating properties and compared to the literature values in order to demonstrate the comparability of the microfluidics-based platform to other INP measurement instrumentation and techniques.

### Homogeneous freezing of water

In the absence of nucleation sites, micron-sized water droplets freeze homogeneously at around − 38 °C on typical laboratory timescales (Riechers et al. 2013; Murray et al. 2010; Atkinson et al. 2016). In the atmosphere, however, clouds are thought to be sensitive to homogenous freezing at higher temperatures (> −35 °C) because clouds are sensitive to a very small number of ice crystals (Herbert et al. 2015). This is important in the Earth’s atmosphere where clouds are able to supercool to temperatures of − 35 °C or even lower (Pruppacher and Klett 1997; Choi et al. 2010; Seifert et al. 2015; Kanitz et al. 2011; Rosenfeld and Woodley 2000; de Boer et al. 2011; Cantrell and Heymsfield 2005) and are therefore susceptible to glaciation even when no INPs are present. As such, the study of the homogeneous phase transition of water to ice is important and has seen a great deal of attention. It has long been known that small volumes of water, either inside a capillary (Sorby 1859) or as droplets on hydrophobic plates (Mousson 1858) and in emulsions (Dufour 1861), can be easily supercooled, and most instances of ice nucleation via the application of microfluidic devices have focussed on, or at least included an investigation of, homogeneous freezing of water droplets (Stan et al. 2009, 2011; Edd et al. 2009; Riechers et al. 2013; Weng et al. 2016).

**Fig. 5:**
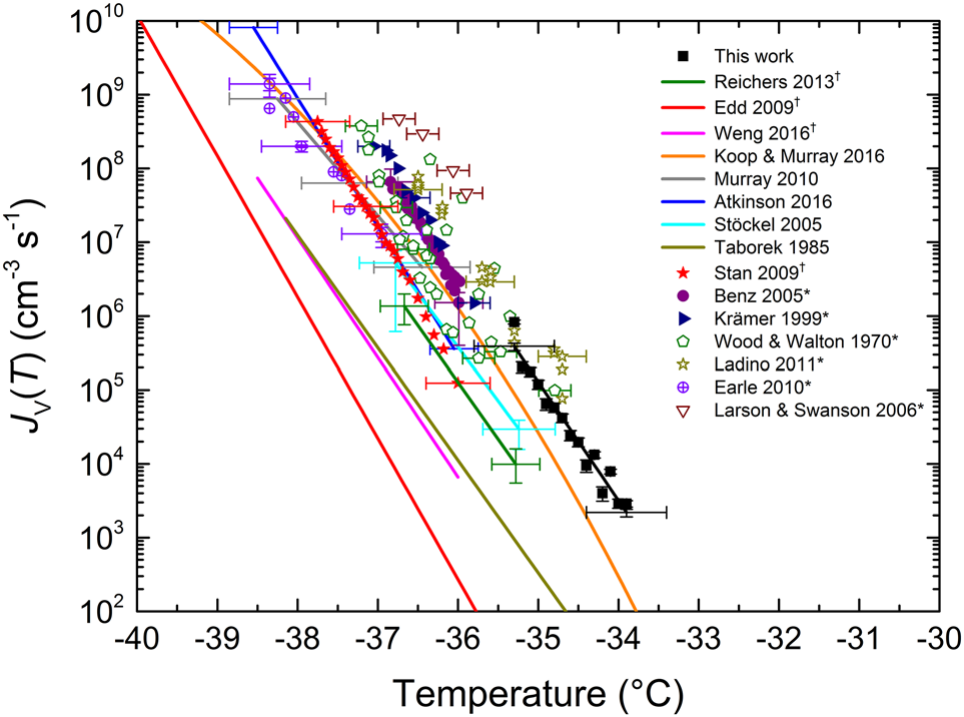
Volumetric nucleation rate coefficients, *J*_V_(*T*), for pure water from our work compared to the literature values. Parameterisations from the literature are shown as solid lines, and where these were not available, the individual data points are plotted. Only a few example error bars are shown for clarity of the plot. Data obtained from the literature via plot digitisation are indicated with an asterisk. Microfluidic examples of homogeneous freezing are indicated with a dagger

In order to test our platform and to provide a blank for our INP results, we studied the homogeneous freezing of 94 ± 3 µm (CV 3%) diameter pure water droplets here. The fraction frozen curve (*T*_50_ = − 34.9 °C) for pure water is shown in Fig. 4a, from which the volume-dependent ice nucleation rate coefficient, *J*_V_(*T*), i.e. the number of nucleation events per unit volume per unit time, was calculated using Eq. (4) (Riechers et al. 2013; Atkinson et al. 2016).

4 $$J_{\text{V}} \left( T \right) = \frac{{ - \ln \left( {\frac{{1 - f_{2} }}{{1 - f_{1} }}} \right)}}{{V\left( {t_{2} - t_{1} } \right)}} = \frac{{ - \ln \left( {1 - \left( {\frac{{\Delta f}}{{1 - f_{1} }}} \right)} \right)}}{{V\Delta t}},$$

where *V* is the droplet volume, *f*_1_ is the fraction frozen at time *t*_1_, and *f*_2_ is the fraction frozen at time *t*_2_. In this equation, the assumption is made that a nucleation event in one droplet is independent of nucleation events in others droplets and that one nucleation event occurs per droplet (Pruppacher and Klett 1997; Broadley et al. 2012). The *J*_V_(*T*) values for the pure water sample were calculated based on a timestep of Δ*t* = 6 s (the time taken for the temperature to decrease by 0.1 °C, with the number freezing events recorded in 0.1 °C bins) and a droplet volume of *V* = 4.42 × 10^−7^ cm^3^. The results are shown in Fig. 5 and provided a fit of: log_10_ *J*_V_(*T*) = − 1.60674·*T *− 51.12734 (*R*^2^ = 0.956, *J*_V_(*T*) error = ± 13%, temperature error = ± 0.5 °C). A selection of literature values of *J*_V_(*T*) are plotted in Fig. 5 for comparison, though more extensive reviews are available elsewhere (Murray et al. 2010; Atkinson et al. 2016). Where possible, parameterisations provided in the relevant papers were used (Edd et al. 2009; Riechers et al. 2013; Weng et al. 2016; Murray et al. 2010; Atkinson et al. 2016; Koop and Murray 2016; Stöckel et al. 2005; Taborek 1985), while in some cases the raw data were kindly provided by the authors (Stan et al. 2009). In the remaining cases (noted in the plot legend with *) (Benz et al. 2005; Ladino et al. 2011; Krämer et al. 1999; Earle et al. 2010; Wood and Walton 1970; Larson and Swanson 2006), the data were extracted from the papers using a plot digitiser (http://arohatgi.info/WebPlotDigitizer).

Our results were found to fit in the same region as the majority of the literature. While our *J*_V_(*T*) values were shifted to higher temperatures than those of other microfluidic platforms (Stan et al. 2009; Edd et al. 2009; Riechers et al. 2013; Weng et al. 2016) and some of the parameterisations, this may be due to the correction factor we applied to compensate for the temperature lag, which increased with cooling, between the cold stage and the sample based on physical measurements. However, the errors quoted in our measurements (± 0.5 °C), largely due to the use of thermocouples, easily overlap the majority of the literature values, and likewise the errors of some other literature sources and parameterisations overlap with our measurements, demonstrating good correlation with published and validated techniques. Interestingly, our *J*_V_(*T*) values were found to closely match with the recent parameterisation that was based on a classical nucleation theory (CNT) in which many of the variables were constrained based on the physical properties of supercooled water and ice (Koop and Murray 2016).

### *Pseudomonas syringae* bacteria (Snomax^®^)

*Pseudomonas syringae* is a gram-negative bacterium that acts as a plant pathogen (Hirano and Upper 2000). Of particular note is that it is a highly efficient ice nucleator (Wex et al. 2015; Maki et al. 1974; Möhler et al. 2007; Vali et al. 1976), a property conferred upon it by the *ina* gene that various ice nucleation active (INA) bacteria contain (Garcia et al. 2012; Hill et al. 2014; Green and Warren 1985). This feature allows it to cause frost damage at relatively high temperatures in plant leaves, thus providing access to the nutrients within (Šantl-Temkiv et al. 2015; Lindow et al. 1982). It is also used commercially in a freeze-dried, non-viable form as a “snow inducer” under the brand name, Snomax^®^, for the production of artificial snow.^4^ Importantly, *P. syringae* is known to be present in the atmosphere (Möhler et al. 2007; Morris et al. 2013b, 2014; Huffman et al. 2013; Després et al. 2012) and has been found in hail (Hill et al. 2014; Michaud et al. 2014), snow (Hill et al. 2014; Šantl-Temkiv et al. 2015; Christner et al. 2008a, b), rainwater (Christner et al. 2008a), and cloud water (Joly et al. 2013). However, while biological sources of INPs such as bacteria, as well as fungal spores and pollen, are known to be present in the atmosphere (Huffman et al. 2013; Pratt et al. 2009; Hoose et al. 2010; Prenni et al. 2009), there is some debate over whether they are present in high enough concentrations to trigger events such as precipitation, particularly compared to other sources such as mineral dusts (Morris et al. 2014). On the other hand, it has been suggested that soil and clay particles may act as carriers of biological INPs, particularly extracts and exudates composed of nanoscale INPs (e.g. proteins), which could potentially outnumber the intact forms (O’Sullivan et al. 2014, 2015, 2016; Schnell and Vali 1976).

**Fig. 6:**
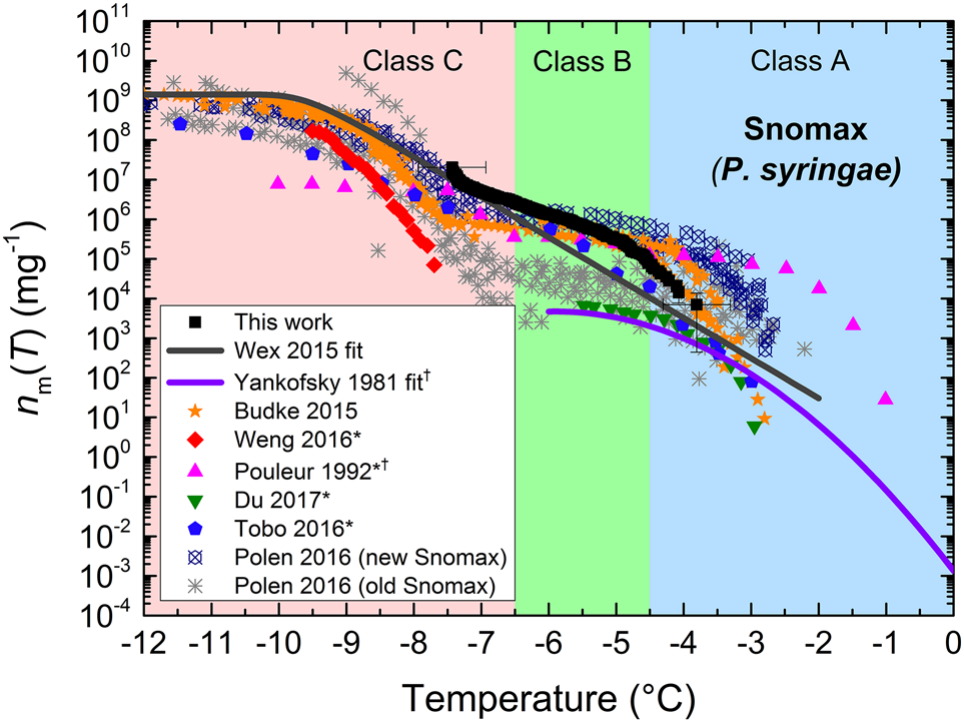
Active site density per mass (*n*_m_(*T*)) of non-viable *P. syringae* in the form of Snomax^®^, with comparison to the literature values. The fit from Wex et al. (2015) was generated via the intercomparison of seven different instruments. The fit from Yankofsky et al. (Després et al. 2012) for *Pseudomonas* bacteria was based on the parameterisation given by Murray et al. (2012). The classes of Snomax^®^ are shown as bands of colour across the relevant temperature range. Literature data obtained via plot digitisation are indicated using an asterisk, while the data from *Pseudomonas* bacteria are indicated with a dagger

Based on the fraction frozen curve for *P. syringae* (Fig. 4a), the median freezing temperature, *T*_50_, was − 6.4 °C, and the droplet diameters were 83 ± 4 µm (CV 4%). Here, we compared our freezing results for *P. syringae* in the form of Snomax^®^ (0.1% w/w, i.e. 1 mg mL^−1^) to those in the literature (both for Snomax^®^ and for strains of *Pseudomonas*, the latter being indicated with †) (Murray et al. 2012; Wex et al. 2015; Tobo 2016; Budke and Koop 2015; Weng et al. 2016; Du et al. 2017; Pouleur et al. 1992; Yankofsky et al. 1981; Polen et al. 2016), based on the calculation of the active site density per unit mass, *n*_m_(*T*), as shown in (3). The results are illustrated in Fig. 6 and, in particular, demonstrate an excellent fit to the Wex et al. (2015) parameterisation for Snomax^®^ that was developed from the intercomparison of multiple instruments within the Ice Nuclei research UnIT (INUIT) project.

Of particular note is the distinct S-shaped curve that was also observed in the experimental data of Wex et al. (2015) (rather than the parameterisation shown here), Polen et al. (2016), and Budke and Koop (2015). Budke and Koop (2015) and Turner et al. (1990) also demonstrated measurable changes in *n*_m_(*T*) for the three classes of Snomax^®^ that trigger ice nucleation in different temperature ranges: Class A (approx. > −4.5 °C), Class B (approx. − 4.5 to − 6.5 °C), and Class C (approx. < − 6.5 °C). In particular, our results indicated that the freezing we observed was associated with Class B and Class C. By comparison, the Snomax^®^ used by Weng et al. (2016) in their cryobiology-focused microfluidic experiments appeared to cause freezing in the Class C regime. Polen et al. (2016) noted that the activity of Snomax^®^ degrades over time, resulting in a significant decrease in droplet freezing temperatures within months of storage, which could explain why our > 1 year-old Snomax^®^ had reduced Class A properties. Beydoun et al. (2016) showed how the concentration of Snomax^®^ studied in immersion mode freezing experiments can affect the *n*_m_(*T*) values below a critical surface area threshold, corresponding to 0.09% w/w in those experiments; hence, we expect that our 0.1% w/w sample was above this critical threshold and so represents the highest *n*_m_(*T*) values expected for the sample.

It should be noted that the dynamic range for our results is smaller than some of the literature results shown, but this is due to the fact that we only studied one Snomax^®^ concentration, while some of the literature examples studied a range of concentrations. However, while we only show a limited dynamic range here, this could easily be extended by testing different concentrations of INPs. It could also be extended by greatly increasing the number of droplets being studied (e.g. to look at thousands of droplets).

Having established that the microfluidics-based platform was capable of measuring biological INPs, we then extended its application to the detection of INPs in fungal and pollen extracts, neither of which have previously been demonstrated using microfluidic set-ups.

### Fungal extract

Some types of fungal spores are well known to nucleate ice (Morris et al. 2013a), including those of the plant pathogenic *Fusarium* species (O’Sullivan et al. 2015, 2016; Huffman et al. 2013; Pouleur et al. 1992; Richard 1996; Hasegawa et al. 1994; Humphreys et al. 2001), and are known to be present in the atmosphere (Morris et al. 2014; Huffman et al. 2012, 2013; Després et al. 2012; Elbert et al. 2007; Ana et al. 2013; Sesartic and Dallafior 2011). Although, as described in *P. syringae* section, their impact on clouds is still under discussion (Ana et al. 2013), it is thought that nanoscale ice-nucleating proteins from the fungi (and other bio-aerosols) can preferentially bind to and confer their ice-nucleating properties upon clay and soil dust that can be lofted into the air (Conen et al. 2011; O’Sullivan et al. 2015, 2016; Schnell 1977; Kögel-Knabner et al. 2008; Schnell and Vali 1972, 1973). With this in mind, we prepared droplets of *F. avenaceum* extract (O’Sullivan et al. 2016) and determined the ice-nucleating activity of the nanoscale INPs, relating it to the available literature for *F. avenaceum* (O’Sullivan et al. 2015; Pouleur et al. 1992; Hasegawa et al. 1994; Humphreys et al. 2001; Seifi et al. 2014). However, it should be noted that such fungal sources of INP are not particularly good as standards or for direct comparison, with the number of INP being dependent upon parameters such as culture media (Humphreys et al. 2001) and age (Richard 1996). Nonetheless, comparisons are provided here to indicate the general temperature and *n*_m_(*T*) ranges that *F. avenaceum* has previously been measured in. The droplet diameter was 86 ± 5 µm (CV 5%), and the median freezing temperature, *T*_50_, was − 12.9 °C.

**Fig. 7:**
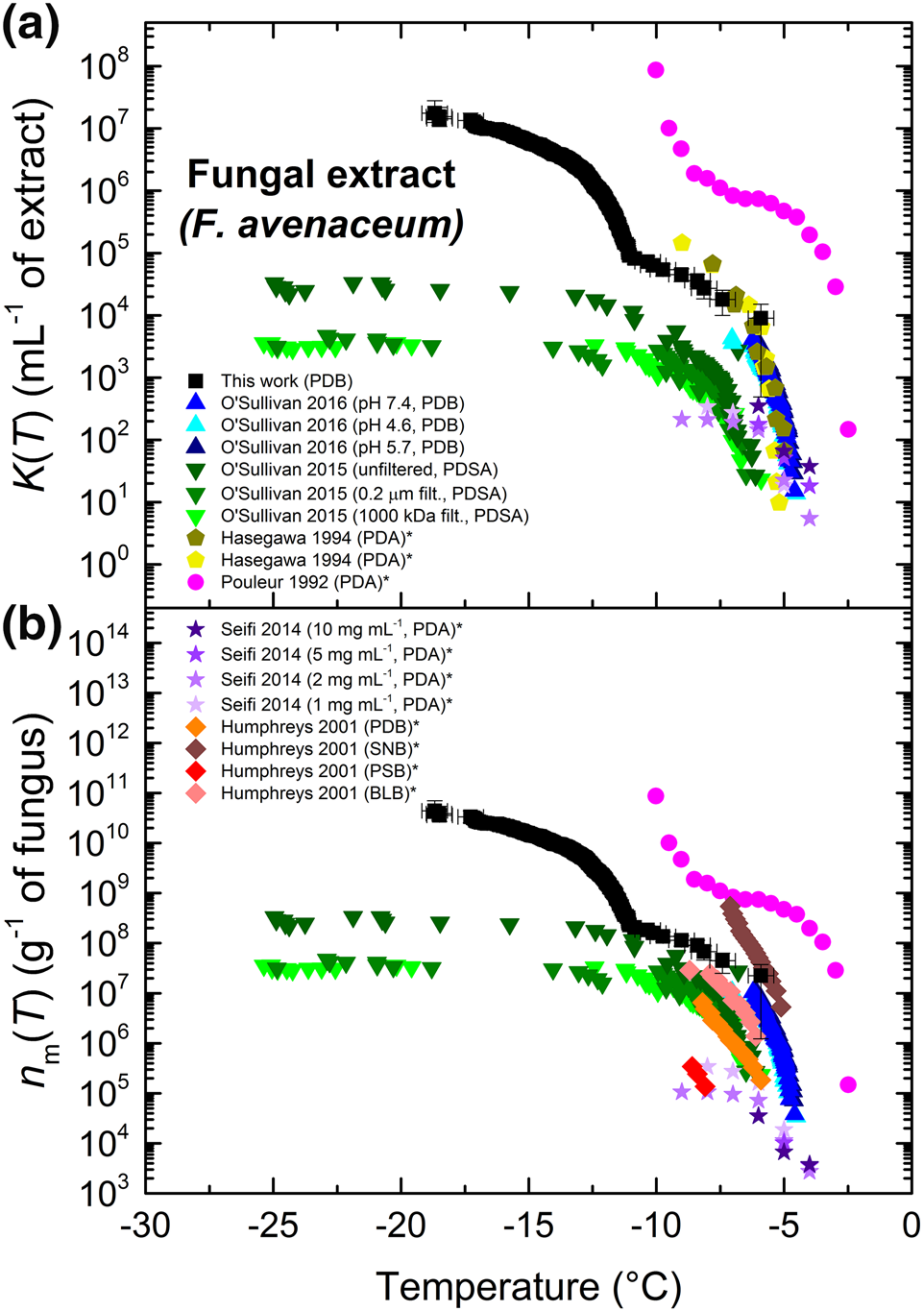
Ice nucleation measurements for an extract of *F. avenaceum* fungus, with comparisons to the literature values. **a** Cumulative number of ice nucleation sites per millilitre of fungal extract (*K*(*T*)) and **b** the active site density per mass of fungus (*n*_m_(*T*)). Literature data obtained via plot digitisation are marked with an asterisk. The abbreviations in the legend refer to the media in which the fungal extract was cultured: *PDB* potato dextrose broth, *PDSA* potato dextrose sucrose agar, *PDA* potato dextrose agar, *SNB* synthetischer nährstoffarmer broth, *PSB* potato sucrose broth, *BLB* banana leaf broth

Figure 7a shows the cumulative number of ice nucleation sites per mL of fungal extract, *K*(*T*), while Fig. 7b illustrates the active site density per gram of fungus, *n*_m_(*T*), calculated based on an initial concentration of 4 × 10^−4^ g mL^−1^ of extract. As can be seen in both plots, the results from the microfluidically generated droplets were in similar ranges compared to the previous literature. [Note that both types of plot are shown since some of the literature data were only available in terms of *K*(*T*) and some in terms of *n*_m_(*T*).] It should also be noted that the fungal material represented here was grown with a variety of different growth media which may explain the differences in the ice-nucleating ability between different samples, as highlighted previously by the data of Humphreys et al. (2001). The results at the higher temperatures are consistent with those of O’Sullivan et al. (2016), who analysed the same fungal extract. The different droplet volumes analysed here (331 pL) and in O’Sullivan et al. (1 µL) yielded data in different temperature ranges. In our data the shape of the curve from around − 11 to − 19 °C is notable, however, as a sharp upward curve upon cooling in this region suggests that there may have been more than one class of ice-nucleating material in the extract. Interestingly, Pouleur et al. (1992) also showed a similar upward curve, albeit of a different shape, at higher temperatures for an unfiltered suspension of *F. avenaceum* at the edge of their data range, possibly suggesting a trend similar to our own. Such comparisons can only be made lightly due to the changing nature of *F. avenaceum* in different conditions, but the results nonetheless suggest that we are capable of detecting fungal-based nanoscale INP.

### Pollen extract

**Fig. 8:**
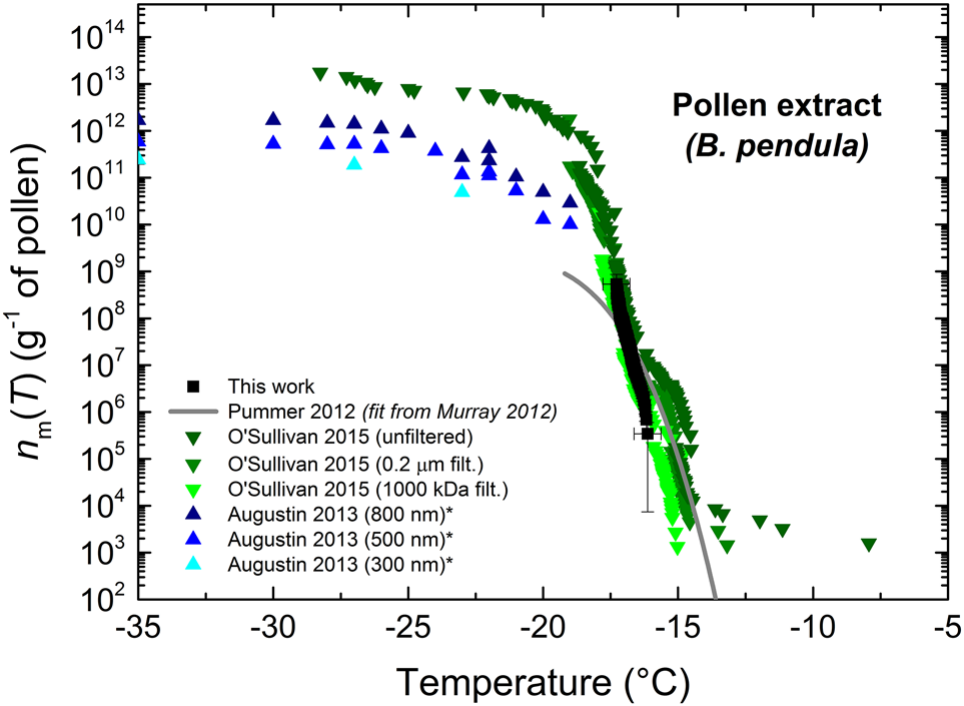
Active site density per mass, *n*_m_(*T*), of wild silver birch pollen (*B. pendula*) extracted into water, with comparisons to values for the same type of pollen found in the literature (data obtained via plot digitisation are marked with an asterisk)

Like some types of bacteria and fungi, pollen is well known as an ice nucleator (O’Sullivan et al. 2015; Diehl et al. 2001, 2002; von Blohn et al. 2005; Augustin et al. 2013; Dreischmeier et al. 2017; Pummer et al. 2012) that is present in the atmosphere (Möhler et al. 2007; Després et al. 2012; Steiner et al. 2015; Sun and Ariya 2006). Pollen contains nanoscale INPs (Hader et al. 2014; O’Sullivan et al. 2015; Augustin et al. 2013; Pummer et al. 2012) that are thought to stem from carbohydrates (Pummer et al. 2012), and a single grain of birch pollen can contain thousands of such INP that are readily released upon contact with water (Augustin et al. 2013). Here, we studied highly active wild silver birch pollen (*Betula pendula*) and compared the freezing results to the literature (O’Sullivan et al. 2015; Augustin et al. 2013; Pummer et al. 2012) in terms of the active site density per gram of pollen, *n*_m_(*T*), as shown in Fig. 8. The fit for the data from Pummer et al. (2012) was calculated from the parameterisation provided by Murray et al. (2012). The droplet diameter was 99 ± 9 µm (CV 9%), i.e. both larger in average size and in variation compared to the other samples. This may have been due to the more viscous nature of the pollen extract that affected the generation of the droplets; in fact, it was observed that using a continuous phase flow rate of 10 µL min^−1^ rather than 20 µL min^−1^ resulted in laminar flow of the pollen extract and oil, an effect not seen with the other INP suspensions where stable droplets could still be formed. The median freezing temperature, *T*_50_, of the pollen extract-containing droplets was − 17.0 °C.

Pollen extract can provide a useful standard due to the steepness of the fraction frozen curve between about − 15 and − 18 °C (e.g. all droplets in this case froze within 1.2 °C). In particular, we prepared the silver birch pollen extract in the same manner and from the same original batch as O’Sullivan et al. (2015), and our *n*_m_(*T*) values were found to closely match that data. Our data were also close to the Pummer et al. (2012) parameterisation (Murray et al. 2012). However, the data from Augustin et al. (2013) for size-selected nanoscale INPs (300–800 nm) were in a different temperature range and so could not be directly compared. With biological sources of INP clearly detectable using the microfluidic set-up, and showing good comparisons to the literature, we further explored the ability of the platform to detect INP from dust sources.

### K-feldspar mineral dust

Wind-blown mineral dusts from desert origins are globally important as atmospheric INP (Tang et al. 2016; DeMott et al. 2003; Connolly et al. 2009; Niedermeier et al. 2010; Niemand et al. 2012; Schnell and Vali 1976; Hoose et al. 2008; Ginoux et al. 2012). The major constituents of this dust are clays (e.g. illite, kaolinite), quartz, and the feldspars (Murray et al. 2012). Of these, the alkali feldspars, in particular potassium (K-)feldspar (KAlSi_3_O_8_) have been shown to be the most efficient ice nucleators and may be the key component in dust in terms of INA (Atkinson et al. 2013; Harrison et al. 2016; Augustin-Bauditz et al. 2014; Zolles et al. 2015; Kiselev et al. 2017), despite their lower contribution to dust mass (~ 3% for K-feldspar; ~ 8% for Na-/Ca-feldspar) compared to the clays (~ 62%) and quartz (~ 16%) (Murray et al. 2012; Atkinson et al. 2013).

**Fig. 9:**
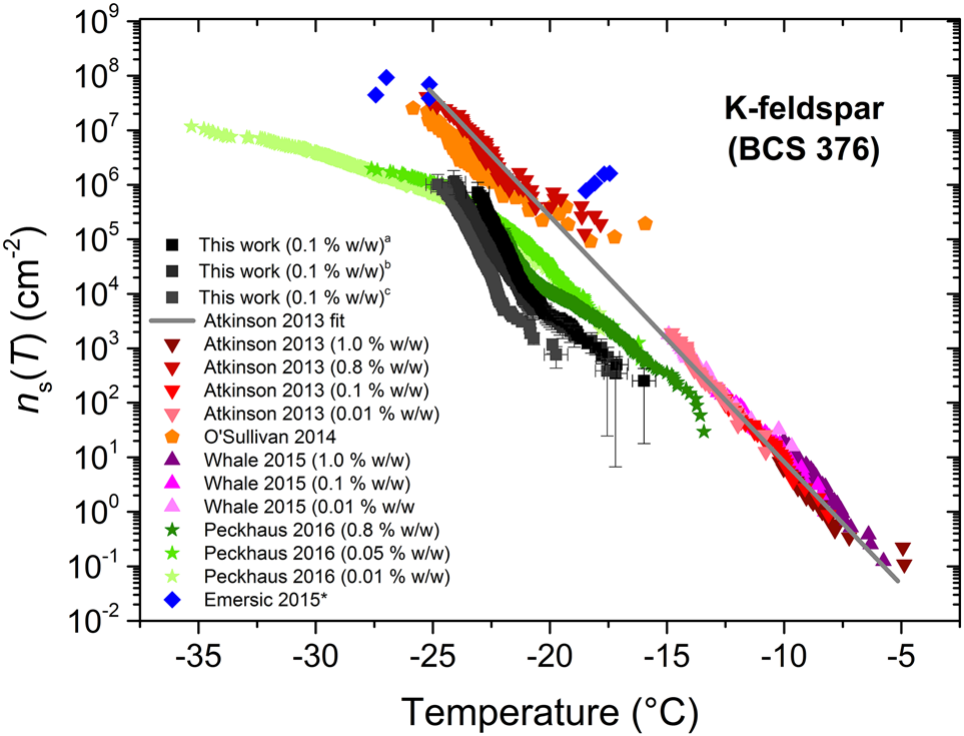
Active site density per surface area, *n*_s_(*T*), measurements for K-feldspar (BCS 376 microcline), with tests performed on samples suspended using three different methods: **a** magnetic stirrer-based mixing, **b** vortex mixing, and **c** overnight rotary mixing. Values from the literature for BCS 376 microcline are provided for comparison. Data obtained via plot digitisation are indicated with an asterisk

Here, we tested a K-feldspar sample (BCS 376 microcline) using 83 ± 7 µm (CV 8%) diameter droplets and compared our results to the BCS 376 literature data (Whale et al. 2015; Atkinson et al. 2013; Peckhaus et al. 2016; O’Sullivan et al. 2014; Emersic et al. 2015) in terms of the ice-active surface density, *n*_s_(*T*), calculated using (2). The *T*_50_ was − 22.8 °C. The mass concentration, *C*_m_, of the K-feldspar sample was 1 mg mL^−1^ (i.e. 0.1% w/w), and the specific surface area, *S*, was 18.6 cm^2^ mg^−1^ (Whale et al. 2015). The results for *n*_s_(*T*) are shown in Fig. 9, with three samples analysed that had been mixed and agitated using different methods to try to break up aggregates and prevent sedimentation in the sample vial: (1) vortex mixing only, (2) mixing using a magnetic stirrer plate followed by vortex mixing immediately prior to the experiment, and (3) mixing overnight on a rotary mixer followed by vortexing immediately prior to the experiment. Most values from the literature, using both 1-µL (via pipetting) (Whale et al. 2015; Atkinson et al. 2013) and 0.4–5.6-pL (via nebulisation; Atkinson et al. 2013; O’Sullivan et al. 2014) volume droplets, fit the Atkinson et al. (2013) parameterisation line (illustrated via a grey line in the plot). Emersic et al. (2015), who used a cloud expansion chamber to look at ice nucleation on a dispersion of feldspar particles, found larger *n*_s_(*T*) values at about − 18 °C, but values of *n*_s_(*T*) consistent with Atkinson et al. (2013) below − 25 °C. Interestingly, our results obtained using 301-pL droplets “stepped off” this line into a lower temperature regime and correlated more with Peckhaus et al. (2016) who employed 215-pL droplets generated via a commercial piezo-driven droplet generator. There was also some variability in our results for the same concentration of K-feldspar. This could have been in part due to the different mixing methods employed that may have been comparatively more or less effective at reducing aggregation and/or sedimentation in the sample vial prior to sampling for the experiments.

We now discuss the possible causes for why the data from Peckhaus et al. (2016) and our new data are lower than the Atkinson et al. (2013) line, and further reasons why the reproducibility was poor in our results. The possibility that there is sedimentation and loss of particles in the system with a corresponding loss of surface area and reduced apparent *n*_s_(*T*) is discussed first. Sedimentation could potentially occur in any part of the microfluidic set-up, including the syringe, the tubing, inside the chip, and in the droplets themselves. We note, however, that sedimentation was not observed in the chip, while visualisation of the flow in the tubing did not suggest sedimentation was occurring there. Sedimentation of the particles, whether agglomerated or not, was observed in the syringe, but the length of the tubing meant that only the particle suspension that was in the tubing at the start of the experiment would be introduced into the chip and therefore the droplets, with the syringe only present to provide the driving force into the chip. Sedimentation in the droplets should not reduce the surface area of particles in the droplets dramatically since the droplets are surrounded by a fluorinated oil phase that the particles should not be able to cross into, i.e. the particles should always remain inside the droplet. It has also been suggested that aggregation of particles within a droplet might lead to an aggregate with dramatically reduced surface area (Emersic et al. 2015). Visual inspection with an optical microscope of particles and aggregates which form over time showed that these aggregates are made up of loosely packed particles where surface area is clearly not reduced by orders of magnitude.

To further address the potential for particles to sediment out during the experiment, we performed a theoretical analysis based on the properties of K-feldspar BCS 376 and the dimensions of the tubing and microchannels. Based on a mean particle diameter of 0.7 µm (from the measurements taken by Atkinson et al. 2013) and a density of 2.65 g cm^−3^, the settling velocity of the K-feldspar particles in water was estimated to be 0.4 µm s^−1^ (Fig. S5 in the ESI). Given the timescale over which droplet generation took place (e.g. ~ 3 min, including 1-min set-up and 2-min droplet collection), together with the inner diameter (380 µm) and length (60 cm) of the aqueous inlet tubing and the applied flow rate (16 µL min^−1^, giving a linear velocity of 2351 µm s^−1^), the particles would settle due to gravity for a vertical distance of 76 µm of the 380-µm tubing diameter (i.e. 20% of the total height). This would correspond to only a 10% loss of the total particle population from the fluid that entered the chip, based on a 0.7 µm particle diameter (Fig. S6a in the ESI). However, Atkinson et al. (2013) showed that while the particle sizes peaked at around 0.7 µm, there was a tail in the distribution up to particles of several 10 s of µm. Therefore, we repeated these calculations for the entire range of particles to calculate the percentage of each particle size population that could be lost in the tubing (Fig. S6a in the ESI), the contribution of each particle size to losses in the total particle population (Fig. S6b in the ESI), and the cumulative losses in the tubing during an experiment (Fig. S6c in the ESI).

These results demonstrated that particle loss due to settling could be as high as ~ 20% of the overall population. Due to the strong dependence of surface area on particle size, however, the loss of such a number of particles could also result in the loss of ~ 50% of the available surface area (Fig. S6). Loss of surface area would affect the ice-active surface density, *n*_s_(*T*), of the K-feldspar, although we note that a loss of 50% of available surface area would yield only a factor two decrease in *n*_s_(*T*). On the other hand, inertial lift forces (comprising the wall interaction force, shear gradient lift force, and secondary-flow drag force; Di Carlo 2009; Martel and Toner 2014) from the tubing wall may have aided to some degree in preventing the particles from settling, and as mentioned above, we did not observe any evidence of particle build-up in the tubing. Therefore, we expect that total particle losses due to settling would not be as high as the values determined for only gravitational settling. It should also be noted that such sedimentation-based losses are not intrinsic to microfluidic systems, but also to a variety of other droplet generation methods such as nebulisation and piezoelectric actuation. The initial sampling of the feldspar sample could also account for some of the variability shown in our *n*_s_(*T*) curves, with the speed and care taken when drawing the sample into the syringe (during which time the sample was no longer being mixed) potentially being crucial in preventing losses of the larger particles or possible agglomerates that would sediment faster. Beydoun et al. (2016) have also demonstrated how *n*_s_(*T*) can change with changing concentration, finding that shifts to lower freezing temperatures when using lower particle concentrations cannot be fully accounted for by normalising to the available surface area. This effect in combination with agglomeration or sedimentation effects could therefore also potentially account for the “stepping off” of our results from the Atkinson et al. (2013) line.

In addition, it should also be considered that the ice-nucleating ability of feldspar may be sensitive to the way in which it is treated prior to freezing. The results from Atkinson et al. (2013) and O’Sullivan et al. (2014), centred around − 20 °C, were obtained by nebulising a suspension to create a fine mist which was then allowed to settle onto a surface and coagulate until droplets of the desired size were obtained. Nebulisation is a more energetic process than using a piezoelectric droplet generator (Peckhaus et al. 2016), or our microfluidic device, and may subject the feldspar particles to stresses which create or expose additional active sites. It is also worth noting that at temperatures lower than − 20 °C the Atkinson et al. (2013) parameterisation over-predicts the activity of airborne desert dusts sampled in the region around Cape Verde (Price et al. 2018). Similarly, Vergara-Temprado et al. (2017) found that their model, based on the K-feldspar Atkinson et al. (2013) parameterisation, under-predicted INP concentrations below about − 20 °C in locations some distance from K-feldspar sources (Vergara-Temprado et al. 2017; Price et al. 2018). This might suggest that the Atkinson et al. (2013) parameterisation is too high at these temperatures by a factor of about 10–100, but is a reasonable approximation at warmer temperatures. Hence, it may be that the results presented here and by Peckhaus et al. (2016) are more representative of K-feldspar in the atmosphere.

Overall, for the ice-nucleating ability of K-feldspar, it is clear that there are discrepancies between different instruments which are not yet explained. We intend to investigate these discrepancies in future work in a more systematic manner, in part because feldspar is thought to be such an important INP type, but also because it may yield a more fundamental understanding of why and how K-feldspar nucleates ice so effectively.

### INP measurements from a rural location in the UK

Having established the capability of the platform for measuring INP in pre-prepared suspensions, we then applied it to the measurement of INPs from atmospheric aerosol samples. In the first instance, samples were analysed from a rural location as part of a larger field campaign. The campaign was undertaken at the University of Leeds Field Research Unit (September–October 2016) (O’Sullivan et al. 2018) and encompassed the deployment of the IcePod, a mobile laboratory housing particle sizing instruments and filters for the measurement of aerosol characteristics and INPs, respectively, in the field. Sampled aerosol particles were washed off the filters and into an aqueous suspension, from which they could be analysed via the µL-NIPI cold stage method (1-µL droplets) (Whale et al. 2015) and scanning electron microscope (SEM) imaging (Ault and Axson 2017), among other techniques. A handful of samples were analysed using the microfluidic platform (with average droplet diameters of 83–87 µm) as an initial test, since this system employs small droplets that allow for detection of the most common (i.e. background level) INPs. Instruments that utilise larger droplets, on the other hand, provide a greater chance of finding rarer but more active INPs.

**Fig. 10:**
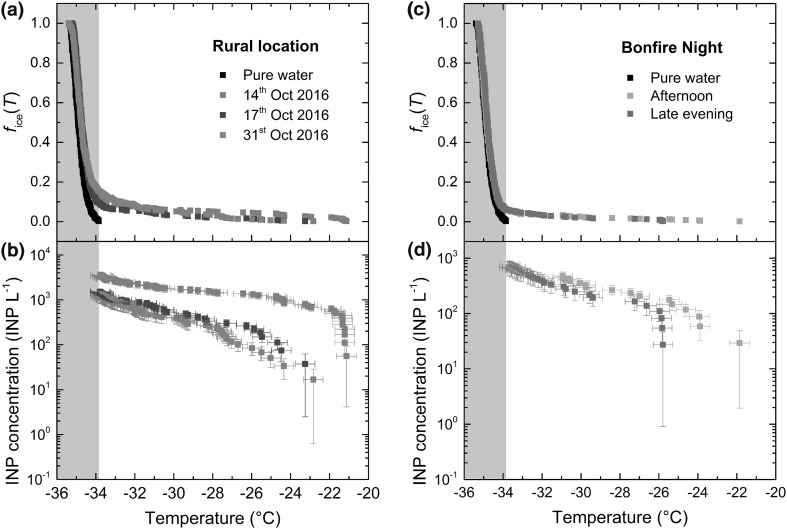
Atmospheric INP measurements from aerosol samples collected during field campaigns: **a**, **b** at a rural location in the UK, collected in October 2016, and **c**, **d** during the UK’s Bonfire Night festival on 5 November 2016. **a** Fraction frozen (*f*_ice_(*T*)) curves for the rural location samples and **b** the corresponding atmospheric INP concentration per litre of sampled air. **c** Fraction frozen curves for the Bonfire Night samples and **d** the corresponding atmospheric INP concentration per litre of sampled air. The shaded regions in the plots below − 33.8 °C indicate the regions where droplets of pure water were observed to freeze; hence, the data from the ambient aerosol data in that region were ignored when plotting the INP concentrations

The major findings from the field campaign itself are available elsewhere (O’Sullivan et al. 2018), but here the fraction frozen curve (Fig. 10a) demonstrated that it was possible to encapsulate atmospheric aerosol particles into microfluidic droplets and freeze them for INP analysis. From the fraction frozen curve, it was possible to estimate the atmospheric INP concentration, [INP], per litre of sampled air using (5):

5 $$\left[ {\text{INP}} \right] = \frac{{ - \ln \left( {1 - f_{\text{ice}} \left( T \right)} \right)}}{V} \cdot \frac{{V_{\text{wash}} }}{{V_{\text{air}} }},$$

where *V*_wash_ is the volume of water used to wash the particles off the collection filter and into suspension, and *V*_air_ is the volume of sampled air. The results are shown in Fig. 10b, with the results below − 33.8 °C removed since the freezing events were then impinging on the region in which droplets of pure water froze. While the number of droplets that froze heterogeneously was low, highlighted by the fact that only ~ 20% of droplets contained INP while the remainder froze homogeneously, INP concentrations compared well with the larger data sets taken from the field campaign (O’Sullivan et al. 2018).

Thus, a microfluidic technique was successfully applied to the measurement of field-sampled INP and was capable of detecting even relatively low, background atmospheric concentrations. This is particularly important given that aerosol sampling in general is very technical due to the ease with which particle losses can be encountered in an unoptimised set-up. Coupled with the rarity of atmospheric INP, the ability to detect INP from sampled aerosol that has been then eluted into suspension represents a crucial result regarding the viability of the technique, particularly given the small volumes of suspension then being employed for microfluidics-based analysis. While the response (i.e., the fraction of droplets that froze heterogeneously) in these initial tests was relatively low, it could be improved in future by sampling the aerosol for a longer period of time and/or at a higher flow rate. Furthermore, the particles were washed off the filters in large volumes of water (5 mL) in order to satisfy the needs of the various other instruments being used. In future studies, however, these volumes could be significantly reduced for application to the microfluidic platform, thereby yielding higher concentrations of particles. While the samples analysed here were frozen and then thawed before use in order to test the platform, the system would be used in future to analyse fresh samples on-site. With this in mind, these preliminary results highlight the potential of the microfluidic platform for deployment in upcoming field campaigns and the potential for routine monitoring of INP concentrations, although further improvements to the procedure and droplet analysis software would be required.

### INP measurements from the Bonfire Night festival (UK)

As a further test of the microfluidic platform for its application to field samples, measurements were taken from samples collected during the annual Bonfire Night festival in the UK. Bonfire Night takes place every November 5 and commemorates the failure of the “Gunpowder Plot” by Guy Fawkes and his co-conspirators in 1605 to blow up the House of Lords and, in doing so, assassinate King James I. The festival yields a very short but intense atmospheric pollution event due to the open burning of assorted fuels (i.e. bonfires) and use of pyrotechnics (Agus et al. 2008; Clark 1997; Moreno et al. 2007; Colbeck and Chung 1996; Singh et al. 2015; Pope et al. 2016). While this festival is UK specific, the atmospheric effects are representative of many other similar global events such as Independence Day in the USA (Liu et al. 1997; Seidel and Birnbaum 2015; Carranza et al. 2001), the Lantern Festival in China (Wang et al. 2007) and Taiwan (Tsai et al. 2012), Las Fallas in Spain (Moreno et al. 2007), Lag BaOmer in Israel (Adler et al. 2011), Diwali in India (Kulshrestha et al. 2004; Ravindra et al. 2003; Chatterjee et al. 2013; Barman et al. 2008), World Cup victory celebrations (Vecchi et al. 2008), and New Year’s Eve (Drewnick et al. 2006; Wehner et al. 2000; Steinhauser et al. 2008), among others. However, few studies have investigated the effect of INP concentrations during such festivals (Ardon-Dryer and Levin 2014), and only a handful have examined other sources of burning biomass, e.g. wildfires, coal-fired power stations, or controlled laboratory burns (Corbin et al. 2012; McCluskey et al. 2014; Prenni et al. 2012; DeMott et al. 2009; Levin et al. 2016; Tan et al. 2014; Umo et al. 2015; Kenneth and Vitaly 2008; Petters et al. 2009; Diehl and Mitra 1998; Schill et al. 2016).

A short campaign was held on 5 November 2016 at the University of Leeds (UK) in order to measure local atmospheric INP concentrations and their relationship to total aerosol and black carbon concentrations, the major findings of which are provided elsewhere (Adams et al. 2018). Aerosol particles collected onto filters throughout the day (approximately 1 sample per hour for 8 h) were washed off and suspended in water for analysis via the µL-NIPI cold stage and the microfluidic platform. The fraction frozen curves, *f*_ice_(*T*), from the microfluidic results (with average droplet diameters of 87–88 µm) are shown in Fig. 10c, with the [INP] values in Fig. 10d, which demonstrate the ability to detect INP even with the very short sampling times. As observed for the results from the rural location campaign, the fraction of droplets which froze heterogeneouslywas low when using the microfluidic platform, with heterogeneous nucleation observed in only up to ~ 10% of droplets, while the remainder froze homogeneously. As described in Sect. 3.8 for the rural location samples, the freezing data below − 33.8 °C were removed in the INP concentration plot (Fig. 10d) since this was the region in which pure water droplets were observed to freeze. Again, however, the INP concentrations agreed favourably with those obtained using the µL-NIPI technique (Adams et al. 2018), with the microfluidic system providing an overview of the more common ice nucleation active particles during the event.

Once again, however, this highlighted the need for longer sampling times or higher sampling flow rates, in addition to lower volumes of water for washing and suspension of particles from the filters, in order to improve the results and limit the number of droplets that freeze homogeneously (due to a low concentration of INP in the final suspension) during tests. Nonetheless, the current microfluidic platform proved capable of measuring atmospherically relevant INP during the rural location and Bonfire Night studies, even at low aerosol concentrations and with a short sampling time, and we intend to use this capability for the measurement of INP in future field campaigns.

## Conclusions

The measurement of a range of atmospheric ice-nucleating particles (INPs) was achieved via the freezing of microfluidically generated droplets. The microfluidic platform enabled the suspension of INPs within monodisperse aqueous droplets that were subsequently cooled on a Peltier stage, allowing hundreds of data points to be collected in the immersion mode freezing regime. The INP characteristics compared well with the literature values for sources that included bacteria, fungal and pollen extract, and mineral dust. The ability to detect INP from atmospheric aerosol, even in low INP conditions, was demonstrated via the analysis of field campaign samples taken at a rural site and during a bonfire event. With this in mind, we intend to deploy the microfluidic platform as part of the IcePod mobile laboratory suite that will enable measurements to be taken around the world at atmospheric observatories and on research ships, and will use the findings to test our state-of-the-art global aerosol models (Vergara-Temprado et al. 2017).

Furthermore, while we designed and fabricated our own microfluidic devices for the development of the platform, other ice nucleation research groups interested in utilising this technology would be able to purchase and use droplet generation chips that are commercially available from several microfluidic chip manufacturers. This would increase the simplicity for the end-user who wishes to use the platform as a monitoring tool, and efforts could be made to establish a “standard design” that could be used by multiple research groups. Looking to the future, further optimisation and integration of the microfluidic set-up will be explored based on the findings and experiences associated with the current set-up and method, in order to combine the droplet generation and freezing steps, as will the potential for direct aerosol sampling into the microfluidic devices (Noblitt et al. 2009; Liu et al. 2016; Mirzaee et al. 2016; Damit 2017). Given the low signals obtained via the aerosol sampling studies presented here, the study of many more droplets would be beneficial to obtain a larger number of INP-containing droplets analysed, for which a continuous flow device such as that of Stan et al. (2009, 2011) may be more suitable for the high-throughput study of 100–1000s of droplets per second. While the current analysis of freezing events is performed manually with the help of a Python program, the measurement of thousands of droplets using that method is not viable, and we are therefore working on improvements to the software to allow automated analysis. This would provide a view to an automated, high-throughput lab-on-a-chip for continuous atmospheric INP monitoring by end-users in the field.

## Supplementary information

The supplementary information contains further details on the fabrication of microwells in glass cover slips, the Peltier-based cryomicroscopy stage, the median freezing temperatures (*T*_50_) of the analysed INP samples, and a theoretical analysis of K-feldspar particle sedimentation.

The data sets for this paper will be made publicly available in the University of Leeds Data Repository (10.5518/334; Tarn et al. 2018).

## Electronic supplementary material

Below is the link to the electronic supplementary material.

Supplementary material 1 (DOCX 19354 kb)

## Footnotes

1 3M™ Novec™ 7500 Engineered Fluid—Product Information, http://multimedia.3m.com/mws/media/65496O/3mtm-novectm-7500-engineered-fluid.pdf, 3M Company. Accessed February 2018.

2 Dolomite Pico-Surf™ product information. https://www.dolomite-microfluidics.com/product/pico-surf-tm-1-10ml-5-in-novec-7500/. Accessed February 2018.

3 Sphere Fluidics Pico-Surf™ product datasheet, http://www.spherefluidics.com/wp-content/uploads/2016/11/Pico-Surf-User-Guide-2016-12-02-V1.pdf. Accessed February 2018.

4 Snomax^®^ International web page. www.snomax.com. Accessed February 2018.
