# Supplementary material for: The study of atmospheric ice-nucleating particles via microfluidically generated droplets
Source: Microfluid Nanofluidics. 2018 Apr 24;22(5):52. doi: 10.1007/s10404-018-2069-x (PMC5915516; doi:10.1007/s10404-018-2069-x)
Supplement: Supplementary file 1 — Supplementary material 1 (DOCX 19354 kb) [file 10404_2018_2069_MOESM1_ESM.docx]

**Electronic Supplementary Information (ESI)**

**The study of atmospheric ice-nucleating particles via microfluidically generated droplets**

Mark D. Tarn,*†^a,b^ Sebastien N. F. Sikora,†^a^ Grace C. E. Porter,†^a,b^ Daniel O’Sullivan,^a^
Mike Adams,^a^ Thomas F. Whale,^a^ Alexander D. Harrison,^a^ Jesús Vergara-Temprado,^a,‡^ Theodore W. Wilson,^a^ Jung-uk Shim^b^ and Benjamin J. Murray*^a^

*^a.^ School of Earth and Environment, University of Leeds, Leeds, LS2 9JT, UK.*

*^b.^ School of Physics and Astronomy, University of Leeds, Leeds, LS2 9JT, UK.*

*‡ Current address*: Institute for Atmospheric and Climate Science, ETH Zürich, Universitätstrasse 16, 8092 Zürich, Switzerland.

† Authors contributed equally to this work.

* Email: b.j.murray@leeds.ac.uk; Tel: +44 (0) 113 34 32887.

* Email: m.d.tarn@leeds.ac.uk; Tel: +44 (0) 113 343 5605.

**Contents**

1. Fabrication of microwells in cover slips **(Fig. S1)**  *Page S2*

2. Peltier-based cryomicroscopy stage **(Fig. S2)**  *Page S3*

3. Droplet diameters **(Fig. S3)**  *Page S4*

4. Median freezing temperatures, *T*_50_, of INP samples **(Fig. S4)** *Page S5*

5. Setting of K-feldspar particles in tubing and potential losses **(Figs. S5-S6)** *Page S6*

6. References *Page S9*

**1. Fabrication of microwells in cover slips**


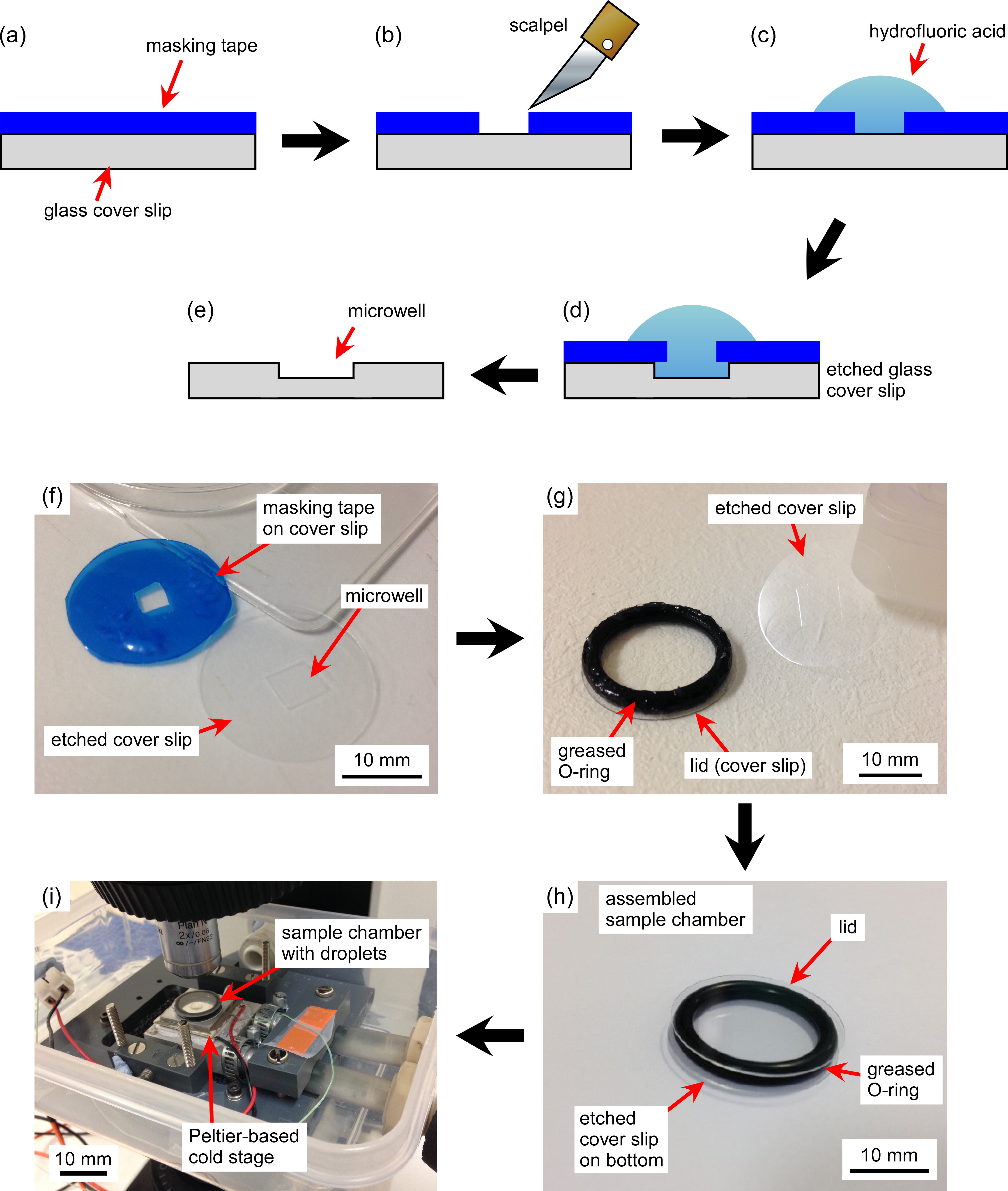


**Fig. S1** (a-e) Fabrication of the microwell cover slips: a glass cover slip coated with industrial masking tape (Intertronics INT600) has a section removed using a scalpel, allowing a solution of 1 % v/v hydrofluoric acid to etch the exposed glass region. (f-i) Photographs showing the preparation of the sample chamber: (f) A masked cover slip alongside a microwell cover slip after etching. (g) A microwell slip alongside a chamber lid, prepared from a second cover slip and an O-ring. (h) Assembled sample chamber, with the lid placed on the microwell cover slip. (i) The sample chamber, containing a suspension of droplets, on the Peltier-based cold stage.

**2. Peltier-based cryomicroscopy stage**


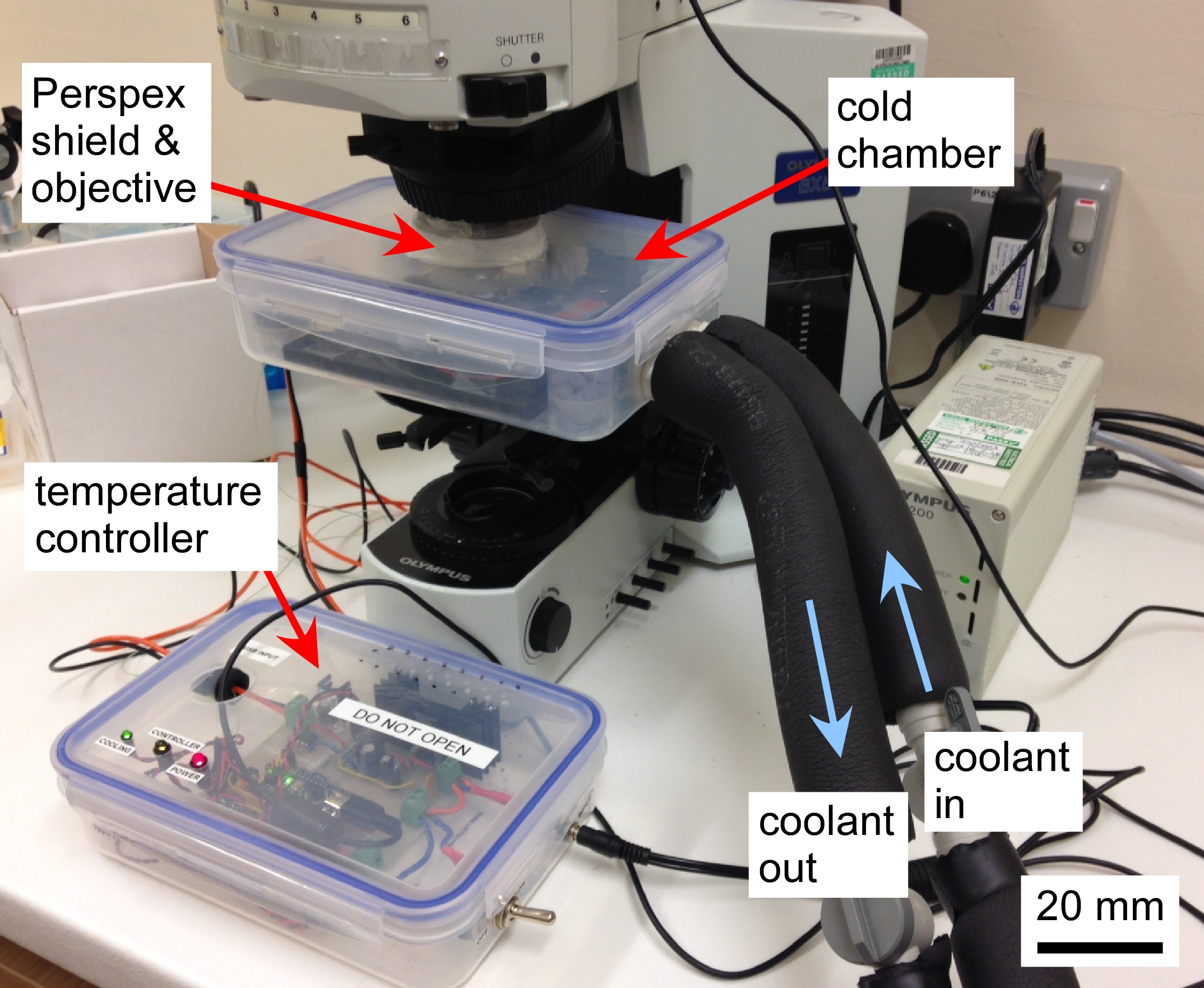


**Fig. S2** Photograph of the cryomicroscopy stage, featuring a cold chamber that housed the Peltier element and liquid heat exchanger. The cold chamber was sealed with the plastic sandwich box, which contained a hole through which the microscope objective was positioned. A Perspex shield was fitted onto the microscope objective via an O-ring, and further sealed against the cold chamber lid with a second O-ring. The liquid heat exchanger was fed via insulated tubing connected to a recirculating chiller unit. The temperature control unit contained an Arduino-based proportional-integral-derivative (PID) controller that was connected to the Peltier and a thermocouple inside the cold chamber.

**3. Droplet diameters**


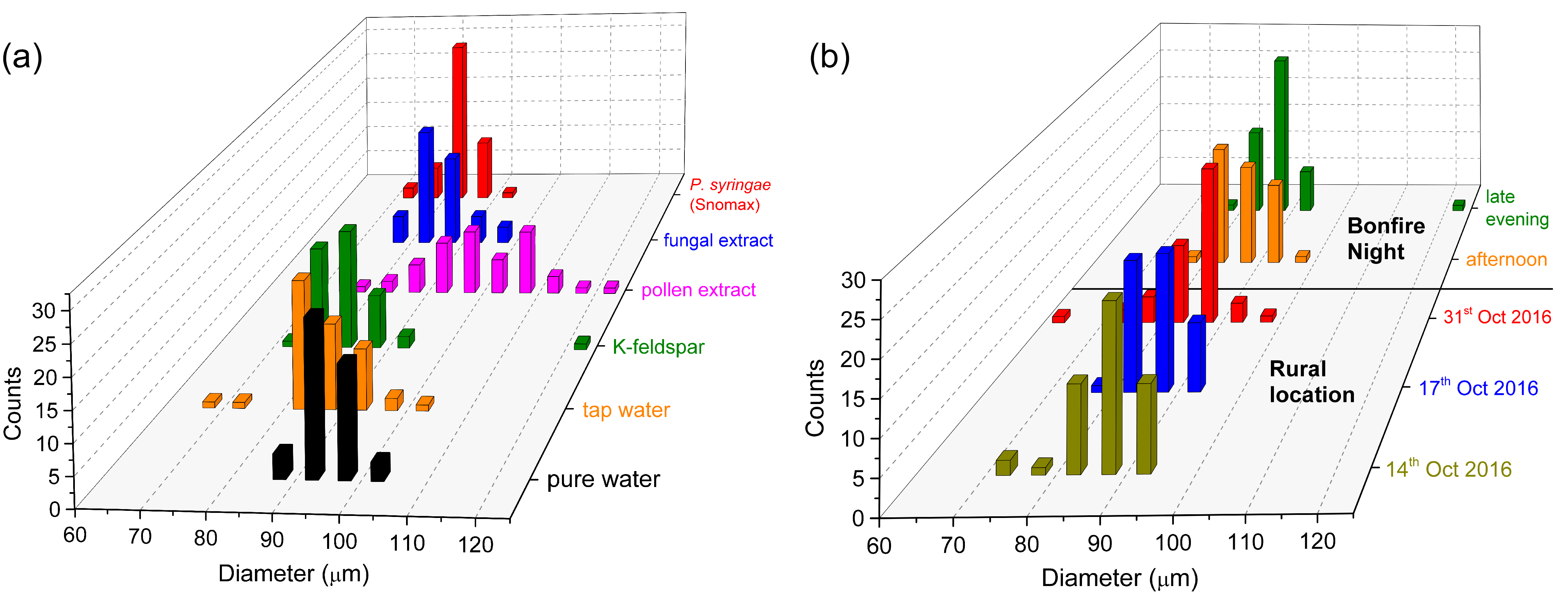


**Fig. S3** Histograms showing the microfluidically generated droplet diameters (*n = 50*) for (a) the range of water and INP samples analysed during validation tests, and (b) the aerosol samples collected during the field campaigns at a rural location and during Bonfire Night.

**4. Median freezing temperatures, *T*_50_, of INP samples**





**Fig. S4** Median freezing temperatures, *T*_50_, for pure water and various atmospheric INP samples analysed using the microfluidic platform.

**5. Setting of K-feldspar particles in tubing and potential losses**





**Fig. S5** Theoretical settling velocities, *u*, of K-feldspar (BCS 376 microcline, density, *ρ*_p_ = 2.65 g cm^-3^) as a function of particle diameter, as determined using Stokes’ law (for Stokes settling):

$u=\frac{2\left( \rho_{p}-\rho_{w} \right)gr^{2}}{9\eta}$ (S1)

where *ρ*_w_ is the density of water at 20 °C (0.998 g cm^-3^), *η* is the viscosity of water at 20 °C (1.002 x 10^-3^ kg m^-1^ s^-1^), *r* is the radius of a K-feldspar particle, and *g* is the acceleration due to gravity (9.81 m s^-2^).

**Fig. S6** Estimated losses of K-feldspar (BCS 376 microcline) particles in the tubing during microfluidic droplet generation experiments, based on their settling velocities shown in Fig. S5. The red line indicates the mean particle diameter of 0.7 μm.^1^ (a) Estimated particle losses in the tubing for each particle diameter. The results indicate, for example, that 90 % of particles that are 3.5 μm diameter would be lost due to settling in the tubing, with particles of larger sizes experiencing losses of >90 % of their population. Particles having the mean diameter (0.7 μm) would experience losses of ~10 % of their population. (b) Contribution of each particle size to the losses of the overall particle population due to settling in the tubing during an experiment. The plots are shown in terms of both the number of particles lost (green bars) and the amount of surface area lost (orange bars), based on the number distribution and surface area distribution of the particles given in Atkinson et al. (Supplementary Information; Supplementary Figure 5).^1^ The plots demonstrate how, for example, the particles with diameters of ~2 μm only contribute a small loss (~1 %) to the overall population, they account for a far more substantial loss of available surface area (~4 %). It should be noted, however, that surface area shown here is based on Atkinson et al.^1^ (*S* = 32.0 cm^2^ mg^-1^), while the K-feldspar used in the microfluidic studies was a more finely ground sample used in Whale et al.^2^ (*S* = 18.6 cm^2^ mg^-1^). (c) Cumulative losses of particles in the tubing with increasing particle diameter, based on the particle number distribution (green line) and surface area distribution (orange line), as determined from the values in (b). It was thus estimated that the maximum loss of particles due to settling in the tubing during a microfluidic experiment would be ~20 % (green line). This loss of particles could therefore represent a potential loss of ~51 % of the available surface area of the original particle population. However, we also anticipate that inertial lift forces (constituting the wall interaction force, shear gradient lift force, and secondary-flow drag force)^3,4^ from the walls of the tubing in the flowing system may help to reduce particle losses caused by settling.





**6. References**

1. J. D. Atkinson, B. J. Murray, M. T. Woodhouse, T. F. Whale, K. J. Baustian, K. S. Carslaw,
S. Dobbie, D. O'Sullivan and T. L. Malkin, *Nature*, 2013, **498**, 355-358.

2. T. F. Whale, B. J. Murray, D. O'Sullivan, T. W. Wilson, N. S. Umo, K. J. Baustian, J. D. Atkinson, D. A. Workneh and G. J. Morris, *Atmos. Meas. Tech.*, 2015, **8**, 2437-2447.

3. D. Di Carlo, *Lab Chip*, 2009, **9**, 3038-3046.

4. J. M. Martel and M. Toner, *Annu. Rev. Biomed. Eng.*, 2014, **16**, 371-396.
